# Supplementary material for: RNA-mediated double-strand break repair by end-joining mechanisms
Source: Nat Commun. 2024 Sep 11;15:7935. doi: 10.1038/s41467-024-51457-9 (PMC11390984; doi:10.1038/s41467-024-51457-9)
Supplement: Supplementary file 1 — Supplementary Information [file 41467_2024_51457_MOESM1_ESM.pdf]

Supplementary Figure 1

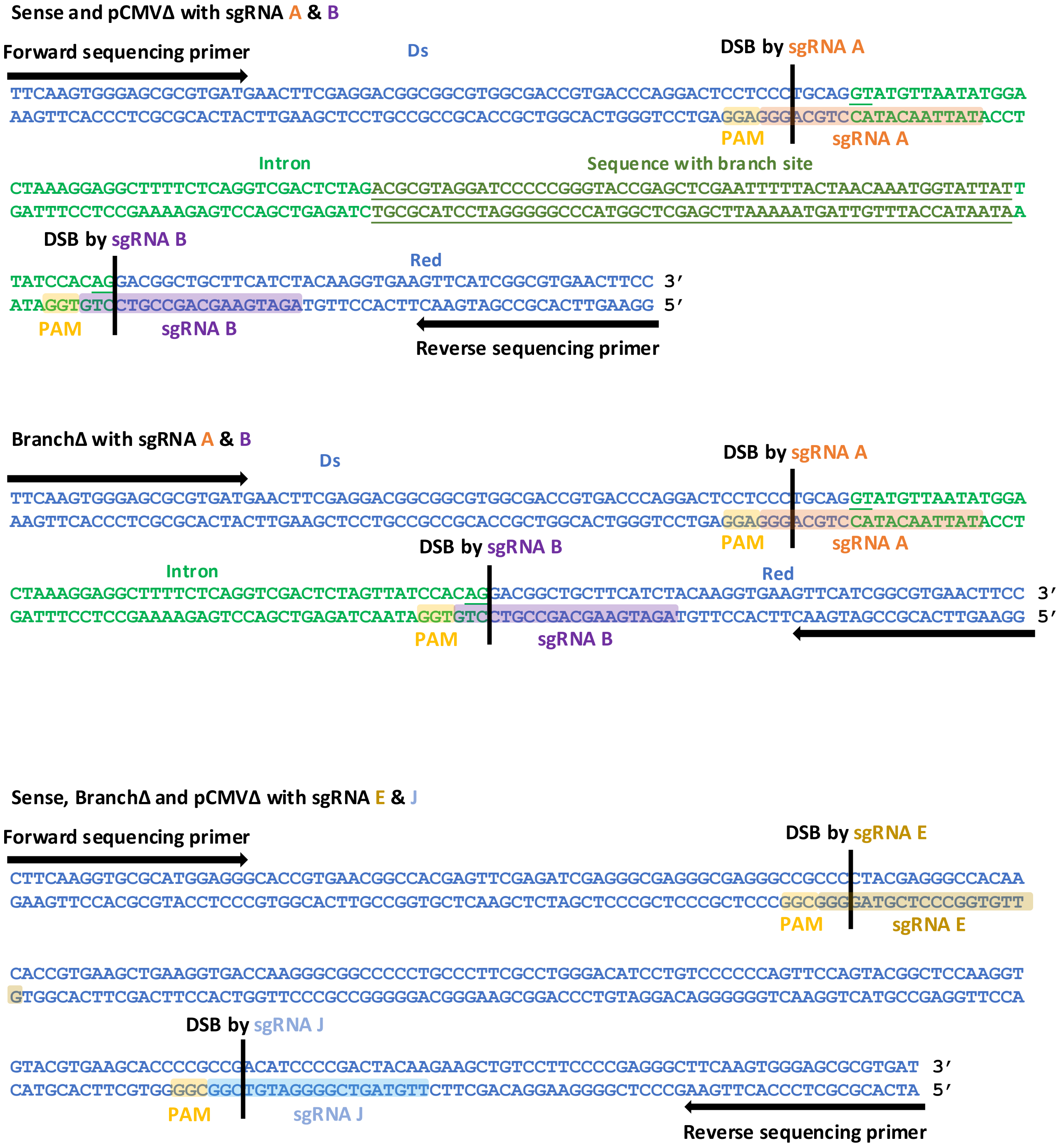

Supplementary Figure 1

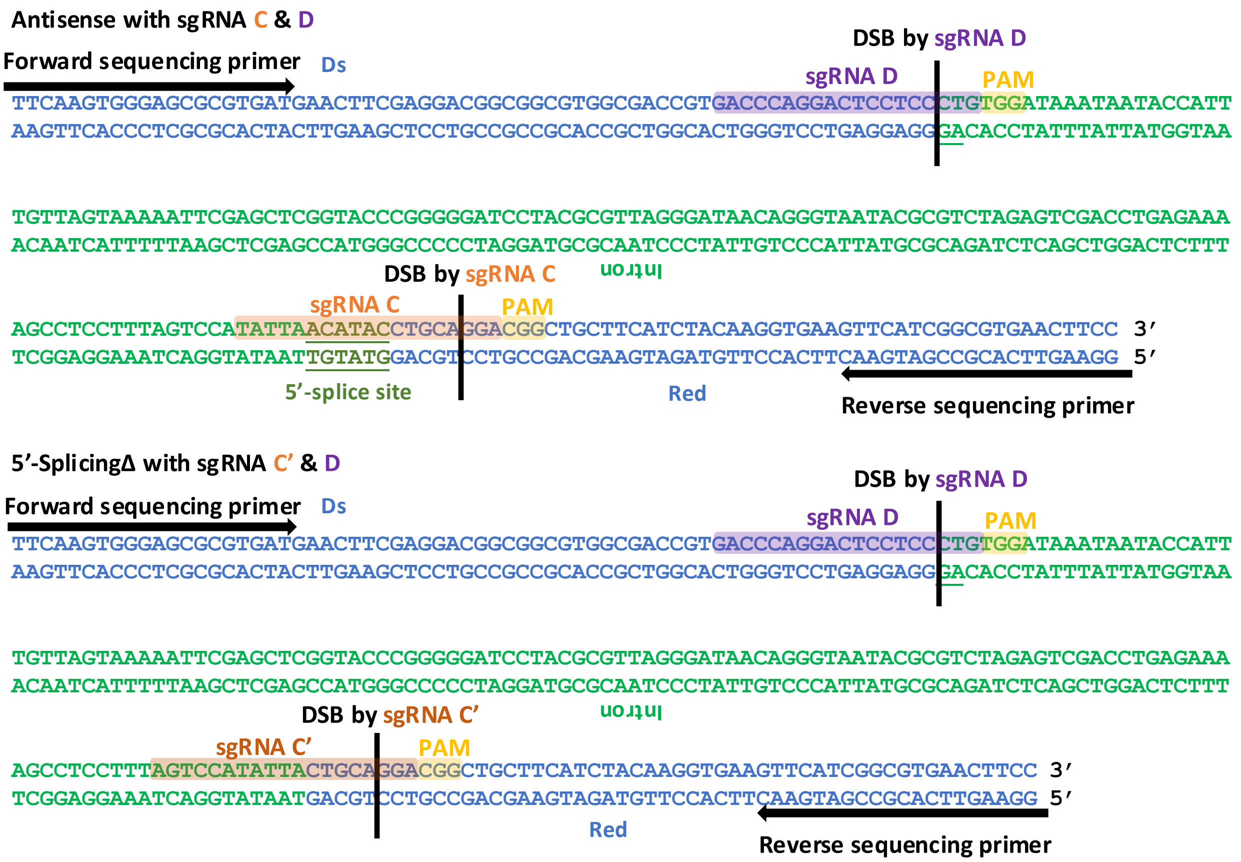

Supplementary Figure 1 | DNA sequence of the *DsRed* loci of the sense and antisense constructs. Blue sequence, exons of the *DsRed* gene; green sequence, intron; underlined green sequence, canonical GT- and -AG splice sites of the intron; underlined dark green sequence, intron sequence containing the branch site (Sense and pCMVΔ) or the 5′-splice site (Antisense). Black arrows, sequencing primers; yellow highlighted sequence, PAM site; orange highlighted sequence, sequence of sgRNA A, C or C'; purple highlighted sequence, sequence of sgRNA B or D; brown highlighted sequence, sequence of sgRNA E; blue highlighted sequence, sequence of sgRNA J. Vertical black bar, site of DSB by sgRNA.

Supplementary Figure 2

a

Plasmid	spliced transcript frequency	non-spliced transcript frequency	alt-spliced transcript frequency	non-canonical alt-spliced frequency	unaligned frequency
Sense	**0.96**	0.0048	0.00043	0.0077	0.026
BranchΔ	0.0034	**0.95**	0.000015	0.010	0.035
pCMVΔ	**0.98**	0.0043	0.000076	0.0032	0.014
Antisense	**0.16**	0.70	0.092	0.0068	0.043
5'-SplicingΔ	0.0042	**0.92**	0.033	0.0075	0.034

b

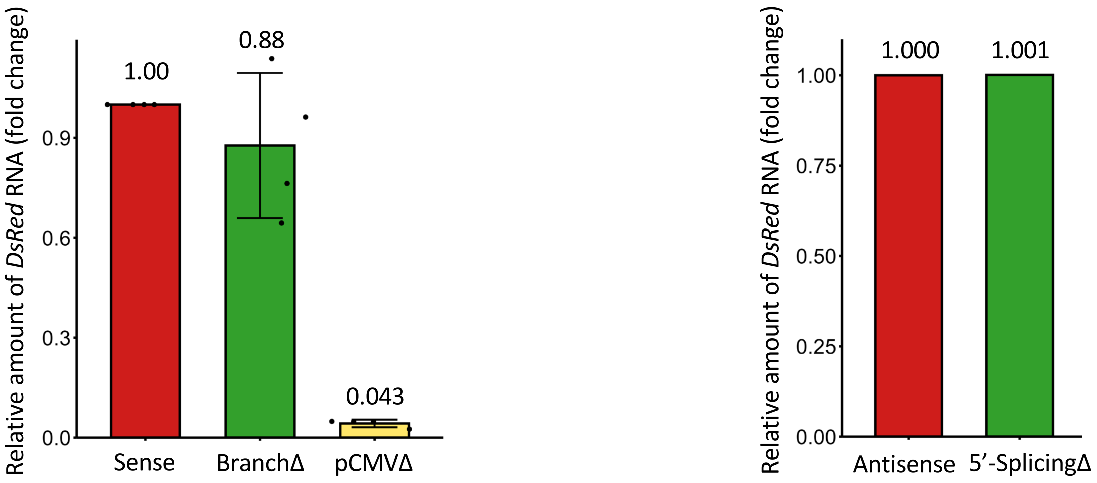

c

Plasmid	spliced transcript frequency	non-spliced transcript frequency	alt-spliced transcript frequency	non-canonical alt-spliced frequency	unaligned frequency
Sense	**0.99**	0.010	0.000	0.000	0.0034
BranchΔ	0.0021	**0.97**	0.015	0.0014	0.014

Supplementary Figure 2 | The RNA transcribed from the BranchΔ and the 5′-SplicingΔ constructs has no intron splicing, and the one transcribed from the pCMVΔ is in low amount.
a, Table of RNA-sequencing results of *DsRed* transcripts from the Sense, BranchΔ, and pCMVΔ, as well as the Antisense and the 5′-SplicingΔ constructs in HEK293T cells. RNA-sequencing reads for each transcript of the different constructs were categorized after alignment to the corresponding DNA sequence (details in Methods). Spliced transcript, a transcript in which the intron was spliced out from the canonical GT---AG-splice sites (see **Supplementary Figure 1**); alt-spliced transcript, a transcript in which splicing happened at alternative GT---AG sites; non-canonical alternative splicing, a transcript sequence that had splicing at sites different from the GT---AG sites. Splicing frequencies were calculated by dividing the number of the spliced-transcript reads by the total number of reads within each RNA-sequencing library, N=1. **b,** Results of RT-qPCR of the *DsRed* transcripts generated from the sense and antisense constructs. Shown on the left is the relative amount of the *DsRed* transcripts from the BranchΔ (green bar) and the pCMVΔ (yellow bar) constructs compared to the amount of the Sense (red bar) transcripts. Shown on the right is the relative amount of the *DsRed* transcripts from the 5′-SplicingΔ construct (green bar) compared to the amount of the Antisense construct (red bar). (Left) Plotted data are the mean fold change ± s.d. of the 4 biological replicates with the individual values shown as dots; N=4. The mean value is shown above each bar. (Right) N=1. **c,** Table of RNA-sequencing results of *DsRed* transcripts from the Sense and BranchΔ constructs in HEK293 cells, N=1. Source data are provided as a Source Data file.

Supplementary Figure 3

a

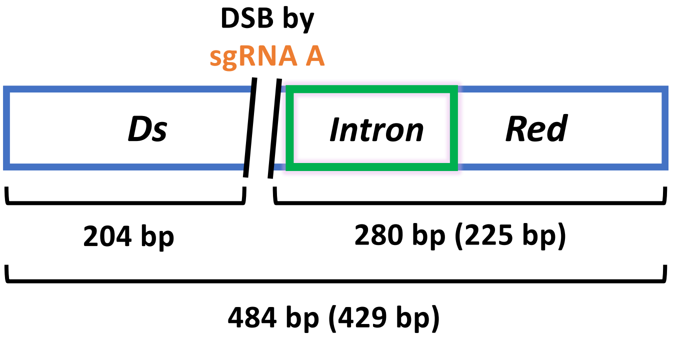

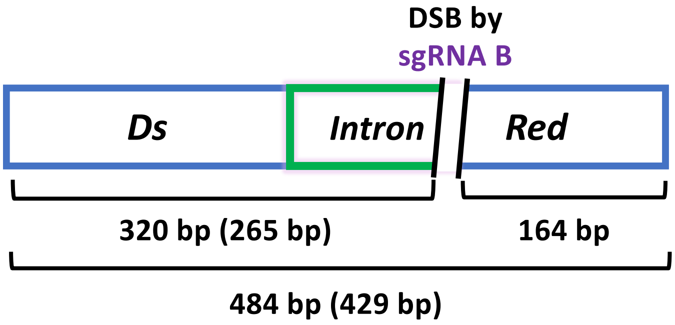

$$\textit{DSB efficiency} = \frac{\frac{204bp + 280bp}{2}}{\frac{204bp + 280bp}{2} + 484bp}$$

(Calculate by molarity)

$$\textit{DSB efficiency} = \frac{\frac{320bp + 164bp}{2}}{\frac{320bp + 164bp}{2} + 484bp}$$

(Calculate by molarity)

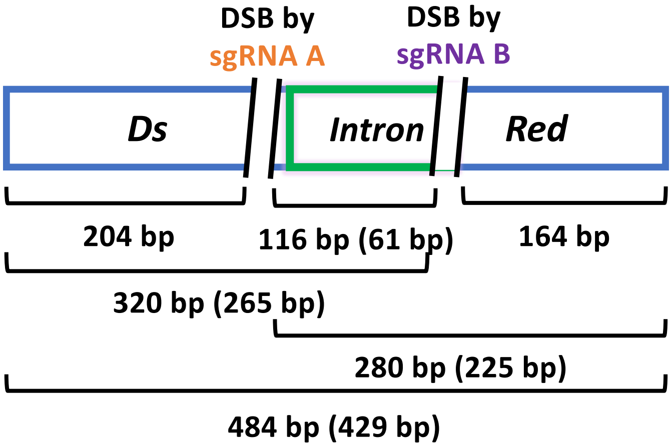

$$\textit{2-DSB efficiency} = \frac{116bp}{116bp + 280bp + 320bp + 484bp}$$

(Calculate by molarity)

**sgRNA A**	1st	2nd	3rd	4th	Average
Sense	**0.936**	**0.939**	**0.952**	**0.938**	**0.941**
BranchΔ	**0.939**	**0.943**	**0.945**	**0.941**	**0.942**

**sgRNA B**	1st	2nd	3rd	4th	Average
Sense	**0.943**	**0.940**	**0.937**	**0.944**	**0.941**
BranchΔ	**0.952**	**0.955**	**0.948**	**0.943**	**0.949**

**2-DSB**	1st	2nd	3rd	4th	5th	6th	7th	8th	9th	10th	11th	Avg.
Sense	**0.890**	**0.891**	**0.883**	**0.879**	**0.891**	**0.882**	**0.884**	**0.885**	**0.876**	**0.882**	**0.889**	**0.885**
BranchΔ	**0.884**	**0.878**	**0.879**	**0.876**	**0.870**	**0.851**	**0.880**	**0.879**	**0.873**	**0.873**		**0.874**

Supplementary Figure 3

b

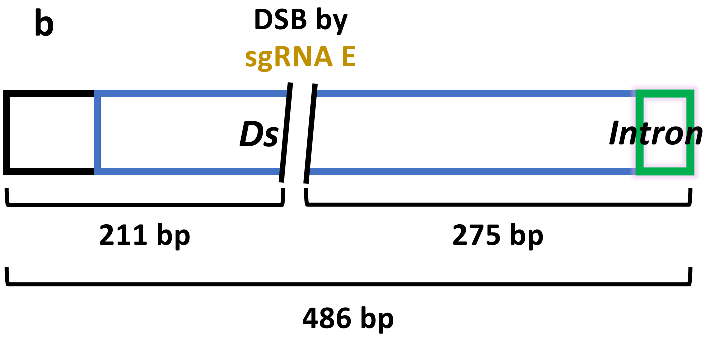

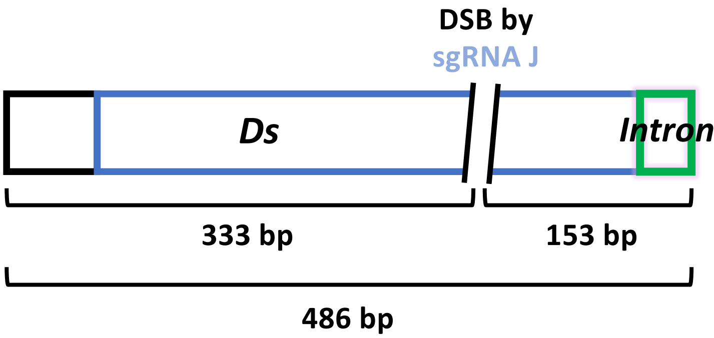

$$DSB\ efficiency = \frac{\frac{211bp + 275bp}{2}}{\frac{211bp + 275bp}{2} + 486bp}$$

(Calculate by molarity)

$$DSB\ efficiency = \frac{\frac{333bp + 153bp}{2}}{\frac{333bp + 153bp}{2} + 486bp}$$

(Calculate by molarity)

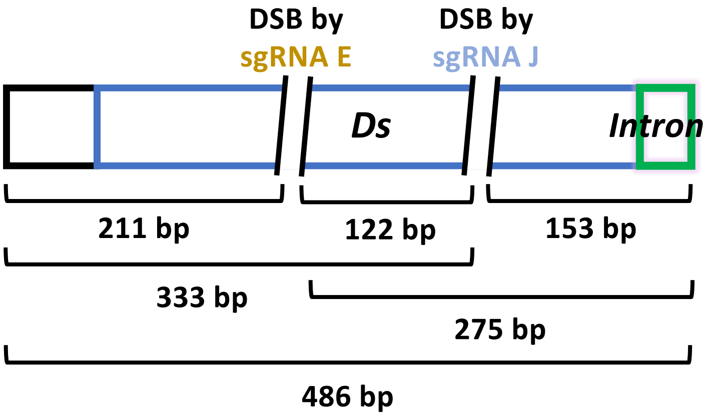

$$2\text{-}DSB\ efficiency = \frac{122bp}{122bp + 333bp + 275bp + 486bp}$$

(Calculate by molarity)

**DSB, sgRNA**	1st	2nd	3rd	4th	Average
1-DSB, sgRNA E	**0.933**	**0.895**	**0.899**		**0.909**
1-DSB, sgRNA J	**0.844**	**0.844**	**0.844**	**0.857**	**0.847**
2-DSB, sgRNA E+J	**0.816**	**0.838**	**0.832**		**0.829**

Supplementary Figure 3

c

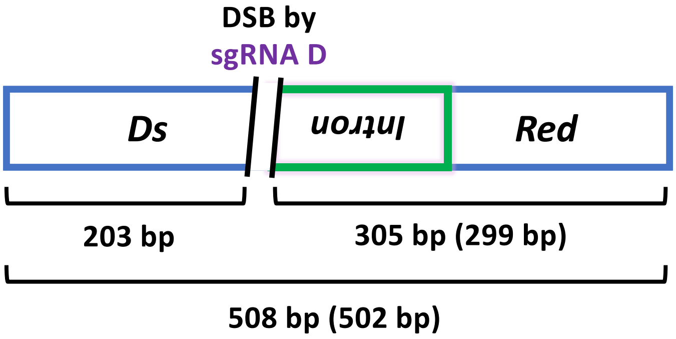

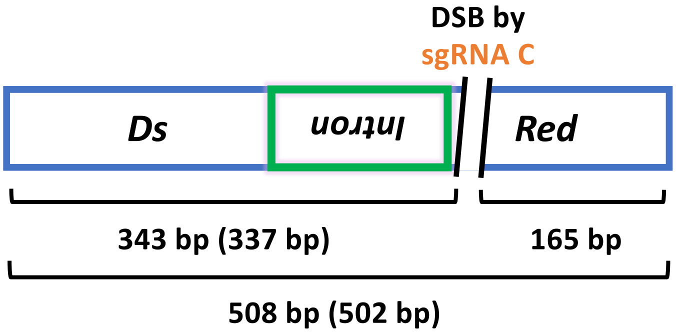

$$DSB\ efficiency = \frac{\frac{203bp+305bp}{2}}{\frac{203bp+305bp}{2}+508bp}$$

(Calculate by molarity)

$$DSB\ efficiency = \frac{\frac{343bp+165bp}{2}}{\frac{343bp+165bp}{2}+508bp}$$

(Calculate by molarity)

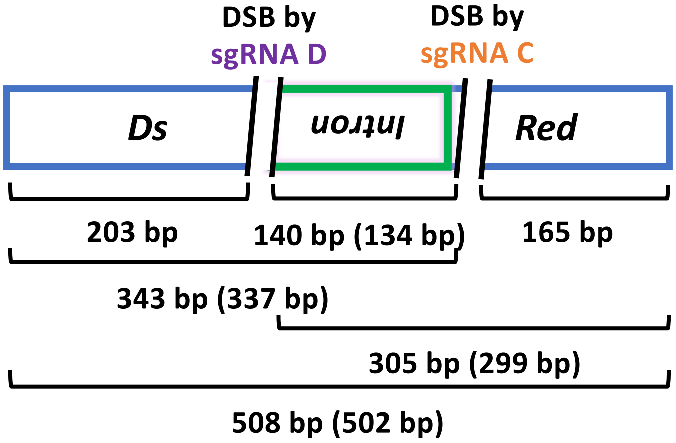

$$2\text{-}DSB\ efficiency = \frac{140bp}{140bp+305bp+343bp+508bp}$$

(Calculate by molarity)

**sgRNA C/C'**	1st	2nd	3rd	4th	Average
Antisense (sgRNA C)	**0.949**	**0.947**	**0.955**	**0.952**	**0.951**
5'-SplicingΔ (sgRNA C')	**0.961**	**0.952**	**0.957**	**0.953**	**0.956**

**sgRNA D**	1st	2nd	3rd	4th	Average
Antisense	**0.836**	**0.824**	**0.846**	**0.849**	**0.839**
5'-SplicingΔ	**0.864**	**0.861**	**0.855**	**0.861**	**0.860**

**2-DSB**	1st	2nd	3rd	4th	5th	6th	Avg.
Antisense	**0.835**	**0.822**	**0.791**	**0.844**	**0.824**	**0.843**	**0.826**
5'-SplicingΔ	**0.835**	**0.853**	**0.846**	**0.860**	**0.854**		**0.849**

Supplementary Figure 3 | Constructs with splicing have similar cleavage efficiency by Cas9 to those without splicing.

Scheme of PCR fragments for **a, b**, the Sense or pCMVΔ construct, and **c,** the Antisense construct used for the *in vitro*-cleavage assay. Cas9 cleavage was done using sgRNA A, B, A and B, E, J and E and J for the sense (Sense/pCMVΔ and BranchΔ) constructs, and by using sgRNA C/C', D or C/C' and D for the antisense (Antisense and 5′-SplicingΔ) constructs. The formulas used to calculate the DSB efficiencies from the molarity of each DNA fragment detected by the Bioanalyzer following cleavage by Cas9 with sgRNA A, B, or A and B for the sense constructs (a), sgRNA E, J or E and J for the sense constructs (b) or sgRNA C/C', D, or C/C' and D for the antisense constructs (c) are shown. The bp sizes in the formulas are those for the Sense/pCMVΔ and BranchΔ (in parenthesis when different) in (a, b), and those for the Antisense and 5′-SplicingΔ (in parenthesis when different) in (c). Results of the *in vitro*-cleavage assay for the sense constructs (a, b), and the antisense constructs (c) are shown in the tables underneath the construct schemes.

Supplementary Figure 4

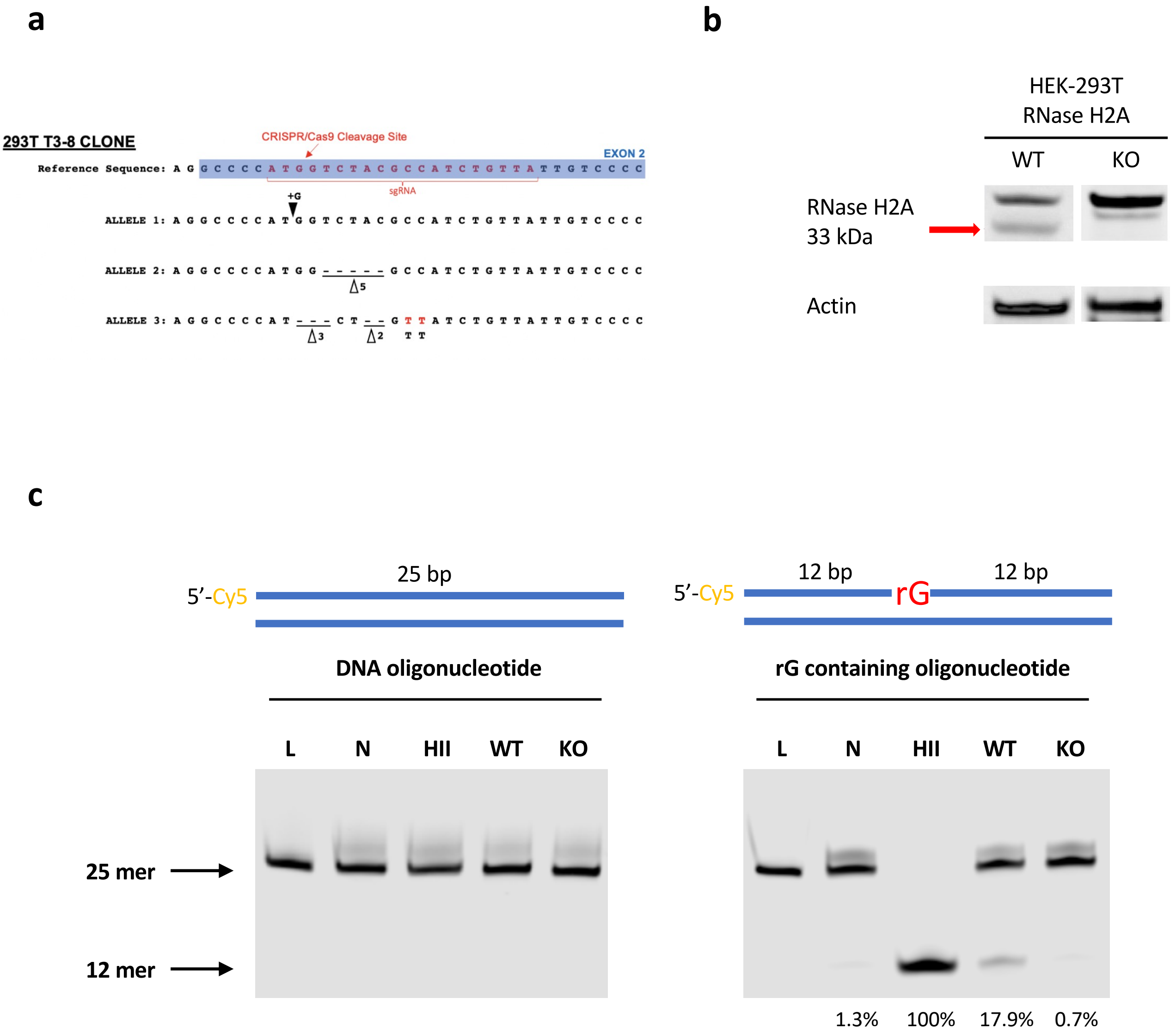

Supplementary Figure 4 | Characterization of RNase H2A KO in HEK-293T cells.
a, Mutated sequences of the *RNASEH2A* alleles from the RNase H2A KO HEK-293T cells. **b**, Result of Western blot for RNase H2A in HEK-293T RNase H2A wild-type and KO cells. **c,** Double-stranded Cy5-labeled 25-mers oligos were used in the *in vitro* cleavage assay to evaluate RNase H2 activity in protein extracts from HEK-293T RNase H2A wild-type and KO cells, schemes shown on the top of each gel image. L, ladder for 25-mers oligonucleotide; N, negative control with the double-stranded oligonucleotide treated by water; HII, *Escherichia coli* RNase HII was used as a positive control cleaving 5′ of the rGMP embedded in the double-stranded DNA oligonucleotides; WT, protein extract from HEK-293T RNase H2A wild-type cells; KO, protein extract from HEK-293T RNase H2A KO cells. The cleavage % is shown underneath the image. Source data are provided as a Source Data file.

Supplementary Figure 5

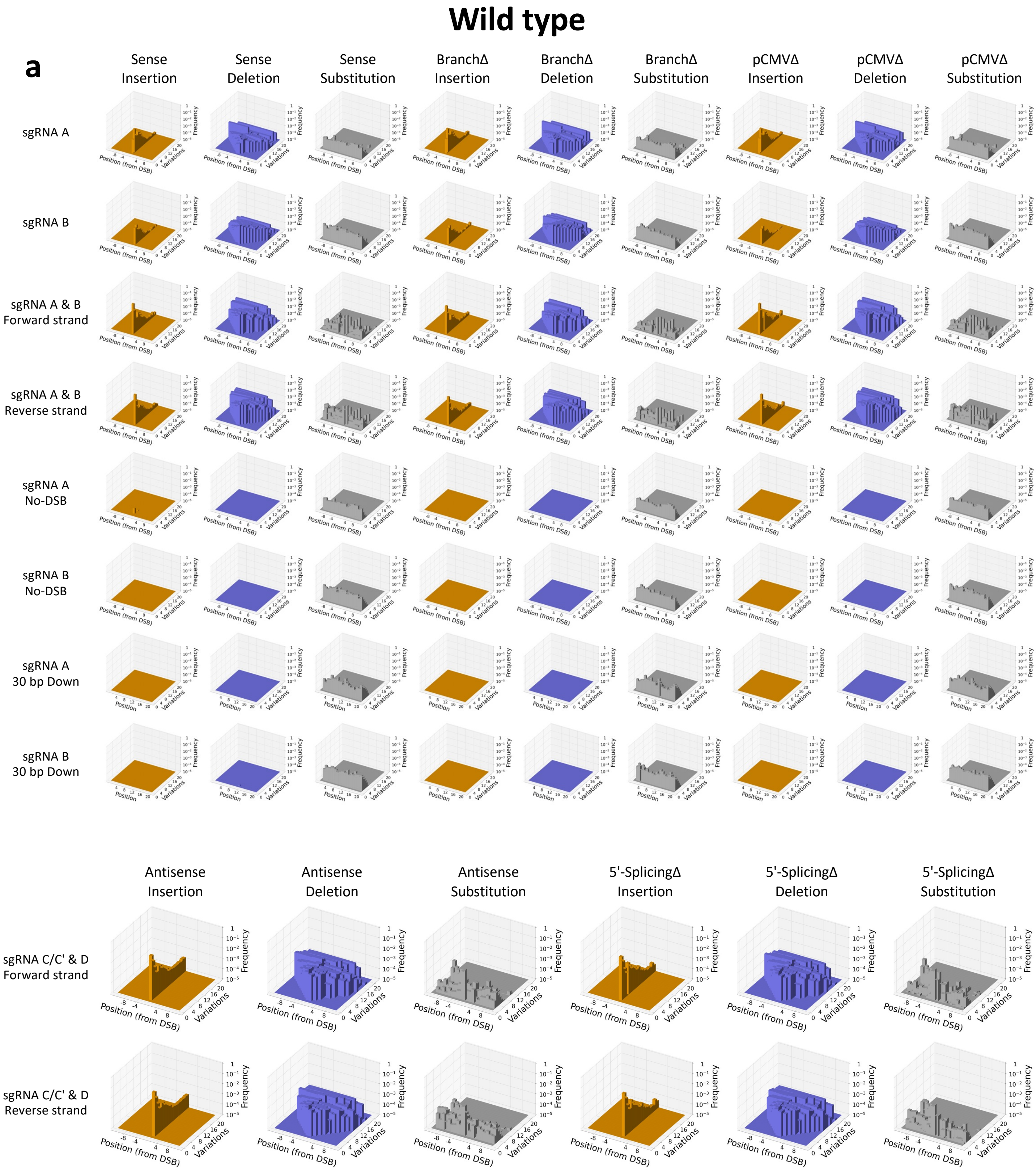

Supplementary Figure 5

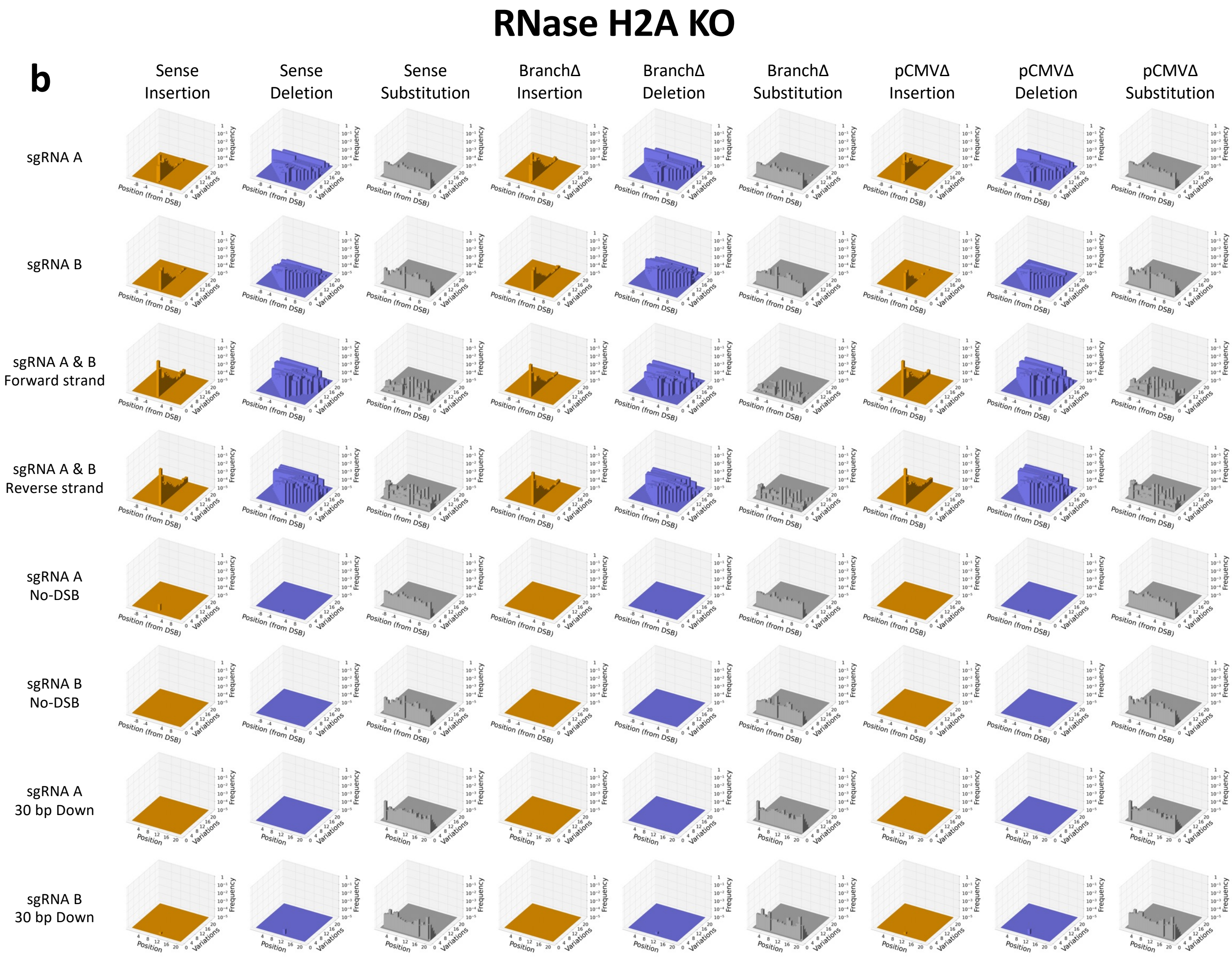

Supplementary Figure 5 | In/dels are sequence variations specific to DSB repair by NHEJ. Variation-position histograms showing the distribution of sequence variations in the 20-bp DSB-sequence windows categorized by the position of the variations and the number of variations. The histograms are arranged in a grid: rows specify the sgRNA(s) used to induce the DSB(s), including the controls (No-DSB and DSB-sequence windows 30 bp downstream from the DSB); columns specify the construct and the type of variation (insertion in orange, deletion in blue, or substitution in gray). The x-axis indicates the position of the variations relative to the DSB site on the reference sequence. Figures with reverse-strand data have their x-axis coordinates reversed so that they correspond to forward-strand coordinates. The y-axis indicates the total number of variations in the DSB-sequence windows. The z-axis indicates the total sum of frequencies (log-scale) of DSB-sequence windows with y variations including position x. If a DSB-sequence window has more than one variation (say k) at the same position (which can only happen if the DSB-sequence window contains insertions), this window contributes k-fold to the corresponding z-value. The x, y, z axes have been limited to the ranges $[-10, 10]$, $[0, 20]$, and $[10^{-5}, 1]$, respectively, and values outside these ranges have been cropped. **a,** Variation-position histograms for experiments conducted in the wild-type cells, and **b,** in the RNase H2A KO cells.

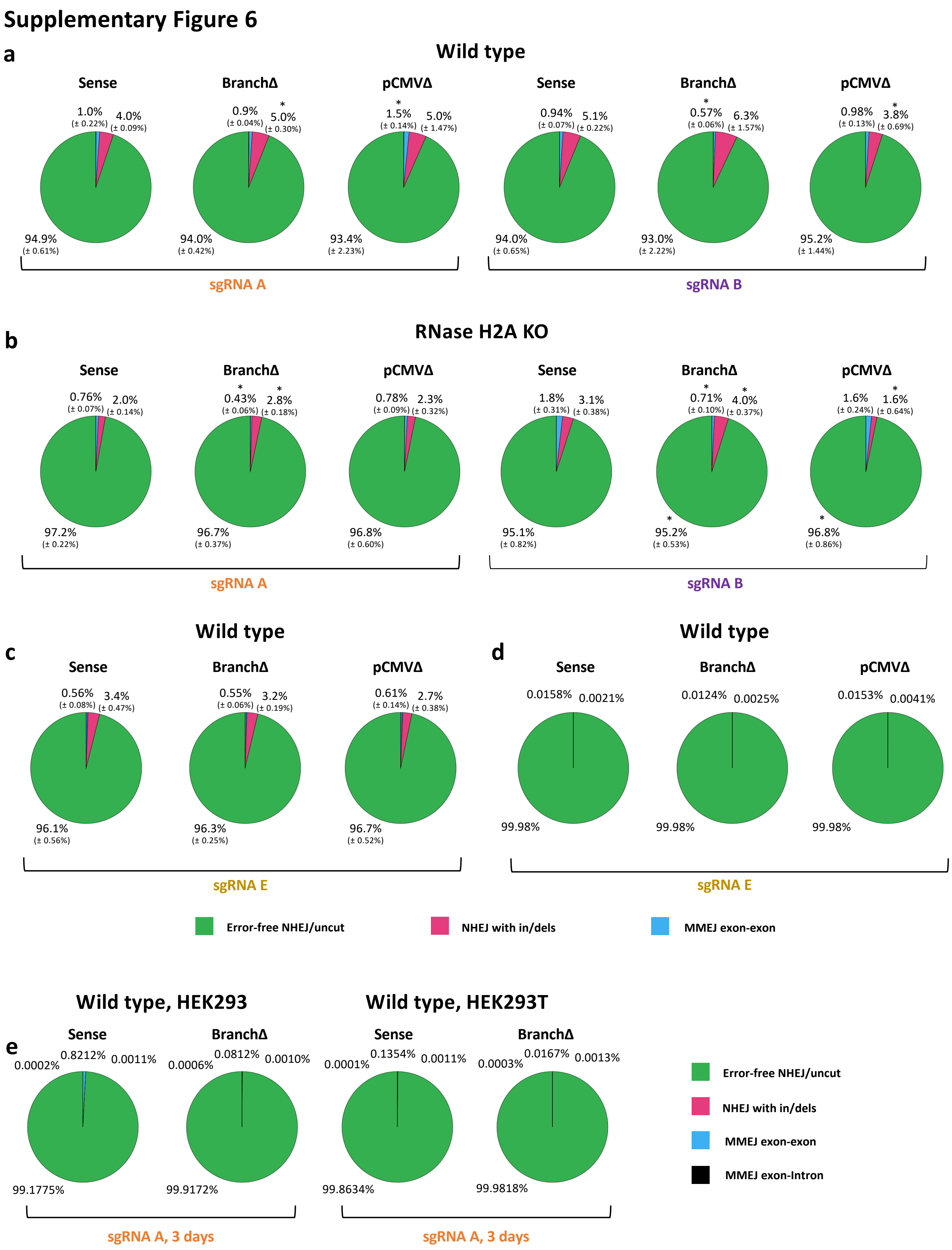
Supplementary Figure 6
a
Wild type
Sense
BranchΔ
pCMVΔ
Sense
BranchΔ
pCMVΔ
1.0% (± 0.22%)
4.0% (± 0.09%)
94.9% (± 0.61%)
0.9% (± 0.04%)
* 5.0% (± 0.30%)
94.0% (± 0.42%)
* 1.5% (± 0.14%)
5.0% (± 1.47%)
93.4% (± 2.23%)
0.94% (± 0.07%)
5.1% (± 0.22%)
94.0% (± 0.65%)
* 0.57% (± 0.06%)
6.3% (± 1.57%)
93.0% (± 2.22%)
0.98% (± 0.13%)
* 3.8% (± 0.69%)
95.2% (± 1.44%)
sgRNA A
sgRNA B
b
RNase H2A KO
Sense
BranchΔ
pCMVΔ
Sense
BranchΔ
pCMVΔ
0.76% (± 0.07%)
2.0% (± 0.14%)
97.2% (± 0.22%)
* 0.43% (± 0.06%)
* 2.8% (± 0.18%)
96.7% (± 0.37%)
0.78% (± 0.09%)
2.3% (± 0.32%)
96.8% (± 0.60%)
1.8% (± 0.31%)
3.1% (± 0.38%)
95.1% (± 0.82%)
* 0.71% (± 0.10%)
* 4.0% (± 0.37%)
* 95.2% (± 0.53%)
1.6% (± 0.24%)
* 1.6% (± 0.64%)
* 96.8% (± 0.86%)
sgRNA A
sgRNA B
c
Wild type
Sense
BranchΔ
pCMVΔ
0.56% (± 0.08%)
3.4% (± 0.47%)
96.1% (± 0.56%)
0.55% (± 0.06%)
3.2% (± 0.19%)
96.3% (± 0.25%)
0.61% (± 0.14%)
2.7% (± 0.38%)
96.7% (± 0.52%)
sgRNA E
d
Wild type
Sense
BranchΔ
pCMVΔ
0.0158%
0.0021%
99.98%
0.0124%
0.0025%
99.98%
0.0153%
0.0041%
99.98%
sgRNA E
Error-free NHEJ/uncut
NHEJ with in/dels
MMEJ exon-exon
e
Wild type, HEK293
Wild type, HEK293T
Sense
BranchΔ
Sense
BranchΔ
0.0002%
0.8212%
0.0011%
99.1775%
0.0006%
0.0812%
0.0010%
99.9172%
0.0001%
0.1354%
0.0011%
99.8634%
0.0003%
0.0167%
0.0013%
99.9818%
sgRNA A, 3 days
sgRNA A, 3 days
Error-free NHEJ/uncut
NHEJ with in/dels
MMEJ exon-exon
MMEJ exon-Intron

Supplementary Figure 6

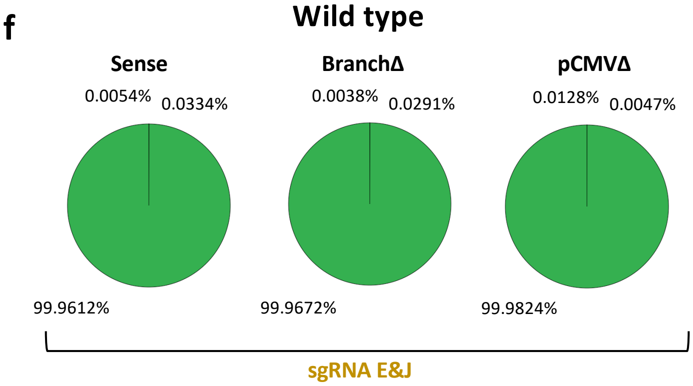

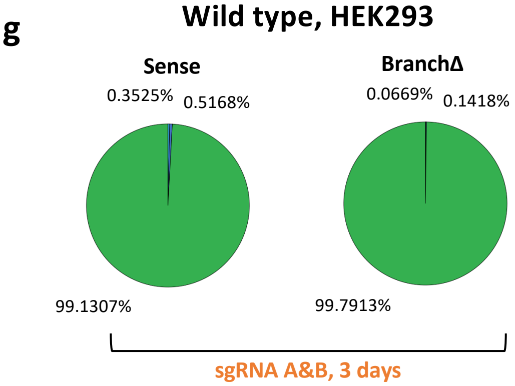

Supplementary Figure 6 | Transcript RNA affects the repair of a DSB in a sequence-dependent manner via NHEJ and MMEJ

Pie charts showing frequencies of sequencing reads in the categories Error-free NHEJ/uncut sequence (green), NHEJ with in/dels (red), or MMEJ exon-exon (blue) following a DSB by the sgRNA A, B or E in the Sense, BranchΔ, and pCMVΔ constructs of wild-type (**a, c**) and RNase H2A KO (**b**) cells. Percentages of the exon-intron MMEJ were 0.07% (wild type, sgRNA A, Sense, ± 0.003%), 0.12% (wild type, sgRNA A, BranchΔ, ± 0.006), 0.07% (wild type, sgRNA A, pCMVΔ, ± 0.013%), 0.03% (wild type, sgRNA B, Sense, ± 0.003%), 0.07% (wild type, sgRNA B, BranchΔ, ± 0.020%), 0.02% (wild type, sgRNA B, pCMVΔ, ± 0.004%), 0.03% (RNase H2A KO, sgRNA A, Sense, ± 0.002%), 0.06% (RNase H2A KO, sgRNA A, BranchΔ, ± 0.008%), 0.03% (RNase H2A KO, sgRNA A, pCMVΔ, ± 0.004%), 0.02% (RNase H2A KO, sgRNA B, Sense, ± 0.002%), 0.03% (RNase H2A KO, sgRNA B, BranchΔ, ± 0.002%), 0.0009% (RNase H2A KO, sgRNA B, pCMVΔ, ± 0.004%). Percentages represent the average of 4 repeats with standard deviations in parenthesis; N=4. *, $P = 0.029$ comparing frequencies of the BranchΔ or the pCMVΔ with those of the Sense construct via the two-tailed Mann-Whitney *U* test. The frequencies have been normalized to only consider sequencing reads classified in one of the three categories (see Methods for an analysis of the unclassified reads). **d, e, f, g,** Pie charts showing frequencies of sequencing reads in each category for No DSB controls without Cas9 expression (see **Method**) in HEK293T cells with sgRNA E (**d**), sgRNA A (**e**, right) or sgRNA E and J (**f**) or in HEK293 cells with sgRNA A (**e**, left) or sgRNA A and B (**g**), N=1. Source data are provided as a Source Data file.

Supplementary Figure 7

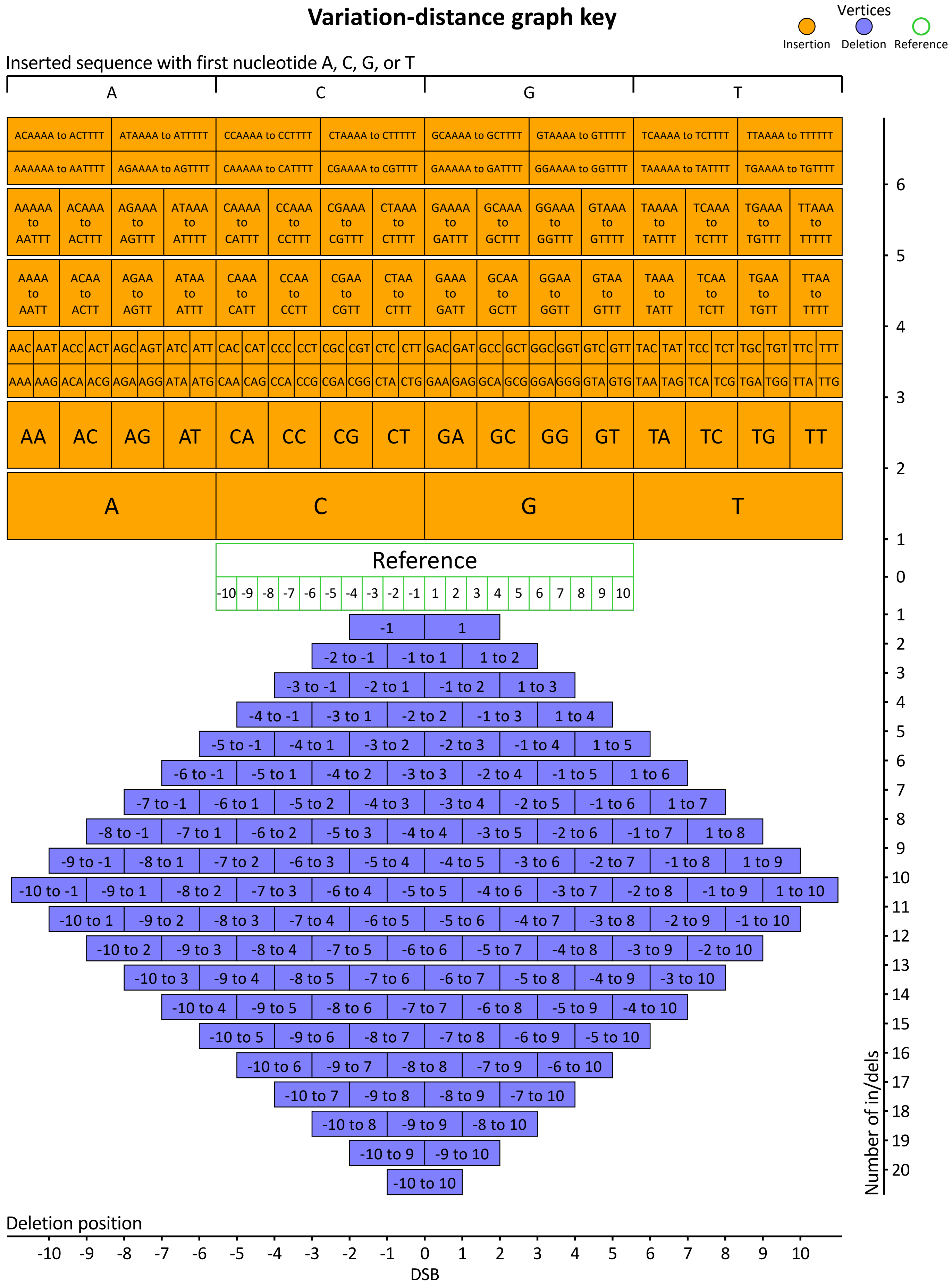

Supplementary Figure 7 | Variation-distance graph key.
Variation-distance graph key showing the placement of the vertices representing the analyzed DSB-sequence windows. Vertex placement depends on both the type of variation (insertion or deletion) and the number of variations of the corresponding DSB-sequence window compared to the reference sequence. Insertion vertices are placed above the reference vertex (center), while deletion vertices are placed below it. For insertions, the alphabetical order of the inserted sequence, from A on the left to T on the right, are indicated by the *x*-coordinate. Each insertion box is labeled with the inserted nucleotides of the DSB-sequence window placed in that location. Insertions of size 3 or more have vertices on multiple lines, staggered vertically to reduce overlap. The *x*-coordinate of deletions indicates the position of the first deleted nucleotide in the given DSB-sequence window, from the most upstream (left-most) to the most downstream (right-most). Each deletion box is labeled with the range of the deleted nucleotide sequence, relative to the DSB site, of the DSB-sequence window placed in that location. The *y*-coordinate indicates the number of variations in the DSB-sequence windows, with higher variations placed further from the reference.

Supplementary Figure 8

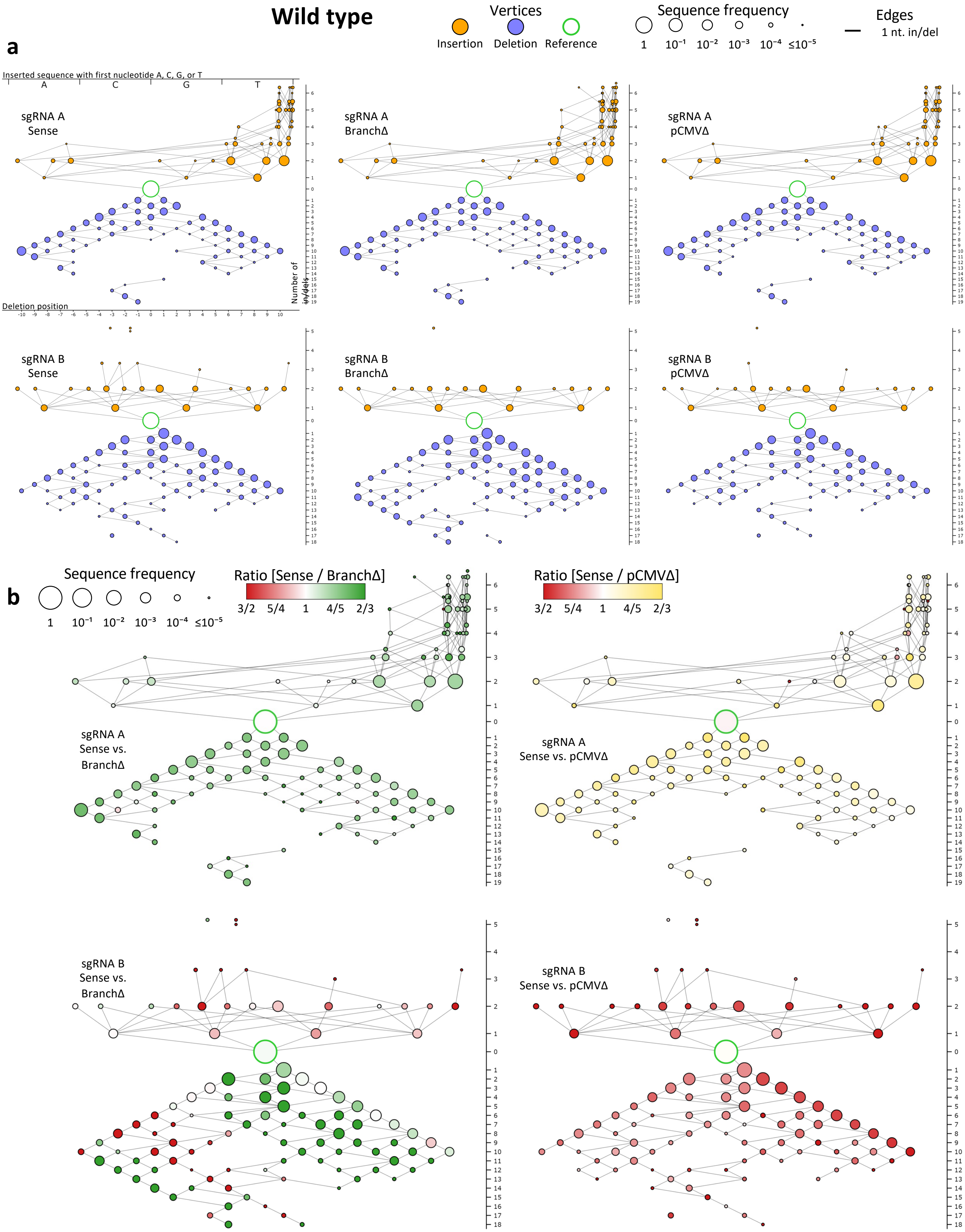

Supplementary Figure 8

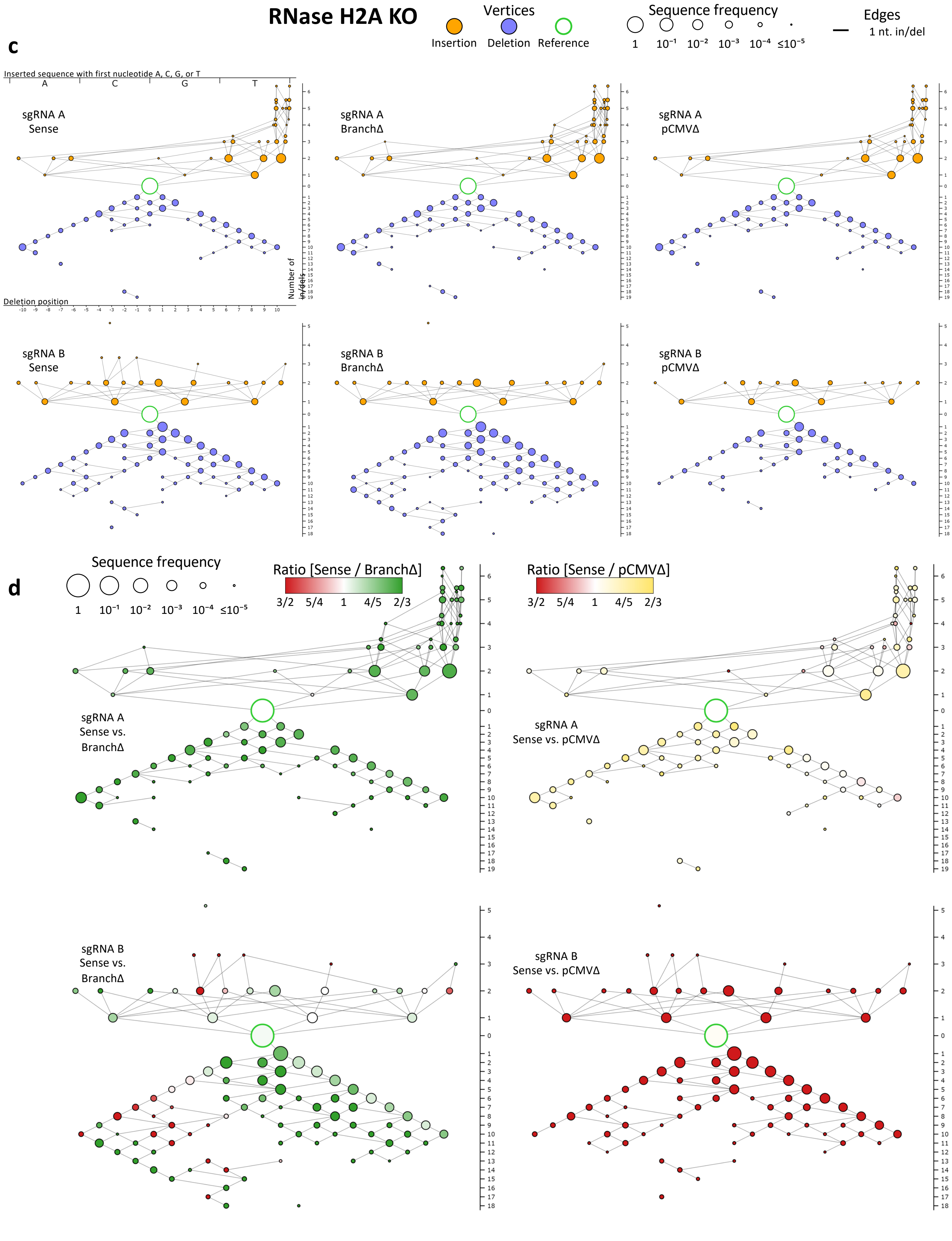

Supplementary Figure 8 | Transcript RNA enhances DSB repair by NHEJ in a sequence dependent manner – all data for the sense constructs.

a, Individual variation-distance graphs illustrating sequence variations within DSB-sequence windows observed after DSB induction by sgRNA A (top) or sgRNA B (bottom) in the Sense, BranchΔ, and pCMVΔ constructs of wild-type cells. An edge between two vertices indicates that the two corresponding DSB-sequence windows differ by a single nucleotide insertion or deletion (in/del). Insertion vertices (orange circles) are placed above the reference vertex (white circle with a green outline), while deletion vertices (blue circles) are placed below it. The vertex size shows the log of the mean frequency of the corresponding DSB-sequence window in the four repeats of the considered experiment. For insertions, the alphabetical order of the inserted sequences, from A on the left to T on the right, are indicated by the *x*-coordinate. Insertions of size 3 or more have vertices on multiple lines, staggered vertically to reduce overlap. The *x*-coordinate of deletions indicates the position of the first deleted nucleotide, from the most upstream (left-most) to the most downstream (right-most). The *y*-coordinate indicates the number of variations in the DSB-sequence windows, with higher variations placed further from the reference. See **Supplementary Fig. 7** for the variation-distance graph key. **b,** Comparison variation-distance graphs of the DSB-sequence windows obtained after DSB induction by sgRNA A (top) or sgRNA B (bottom) for the Sense vs. the BranchΔ construct (left) or for the Sense vs. the pCMVΔ construct (right) of wild-type cells. The vertices represent the same DSB-sequence windows as for the individual graphs while the vertex colors specify the relative frequency in the Sense (red) vs. the BranchΔ (green) construct or in the Sense (red) vs. the pCMVΔ (yellow) construct; the vertex sizes show the log of the maximum of the two mean frequencies of the corresponding DSB-sequence windows in the two analyzed constructs. **c**, Individual variation-distance graphs illustrating sequence variations within DSB-sequence windows observed after DSB induction by sgRNA A (top) or sgRNA B (bottom) in the Sense, BranchΔ, and pCMVΔ constructs of RNase H2A KO cells. **d**, Comparison variation-distance graphs of the DSB-sequence windows obtained after DSB induction by sgRNA A (top) or sgRNA B (bottom) for the Sense vs. the BranchΔ construct (left) or for the Sense vs. the pCMVΔ construct (right) of RNase H2A KO cells.

Supplementary Figure 9

a

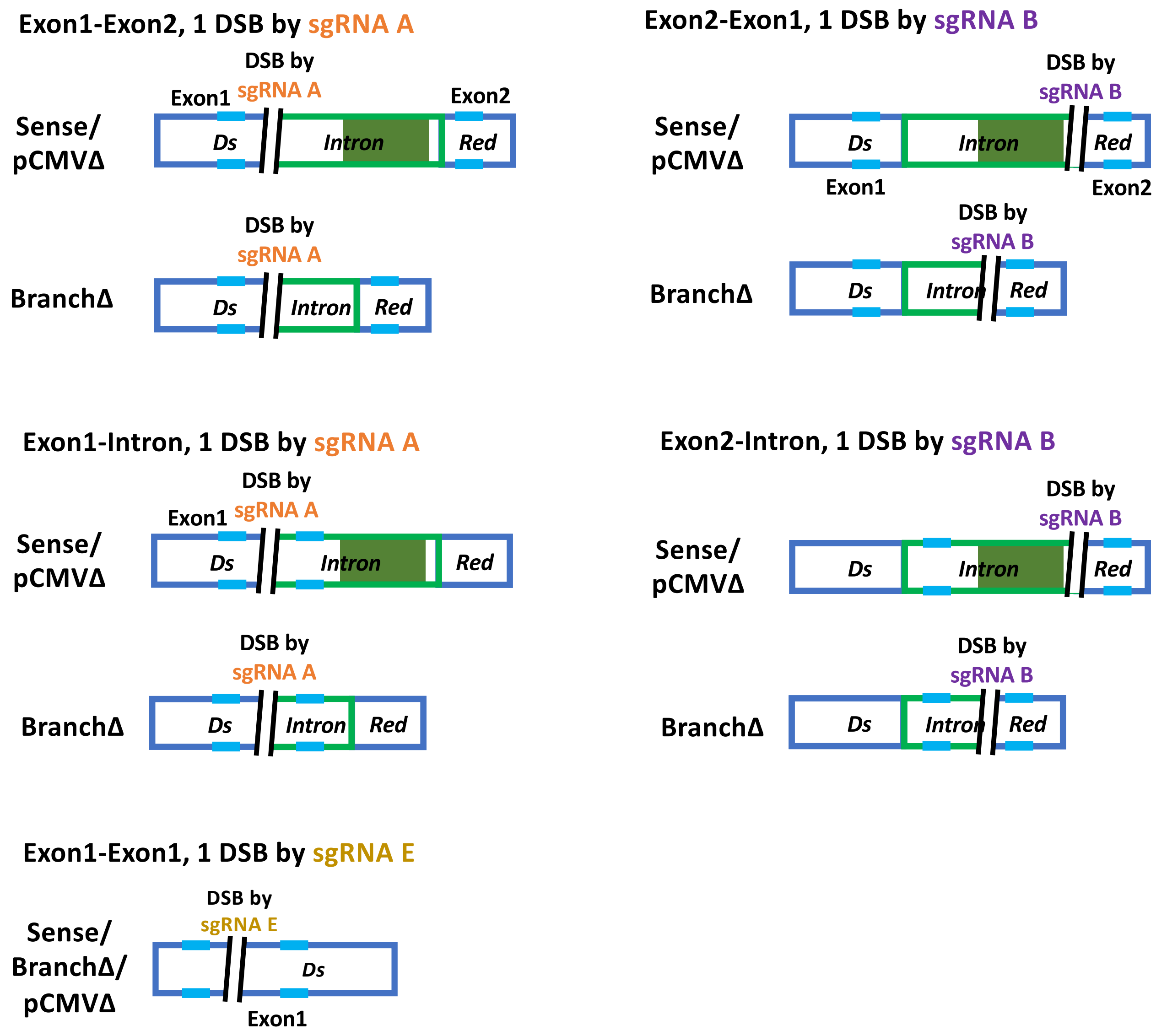

Supplementary Figure 9

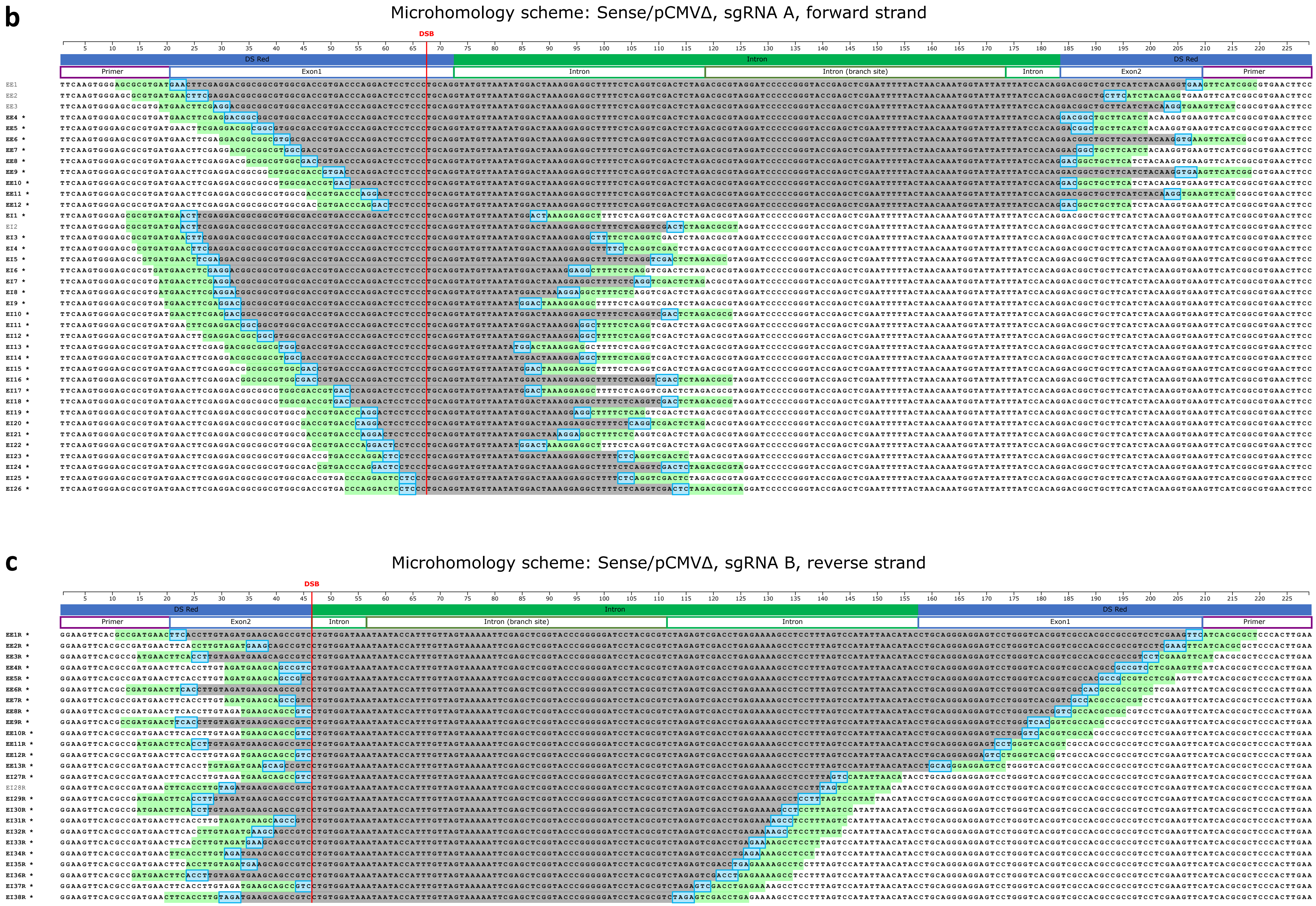

Supplementary Figure 9

d

Microhomology scheme: Sense/pCMVΔ/BranchΔ, sgRNA E, forward strand

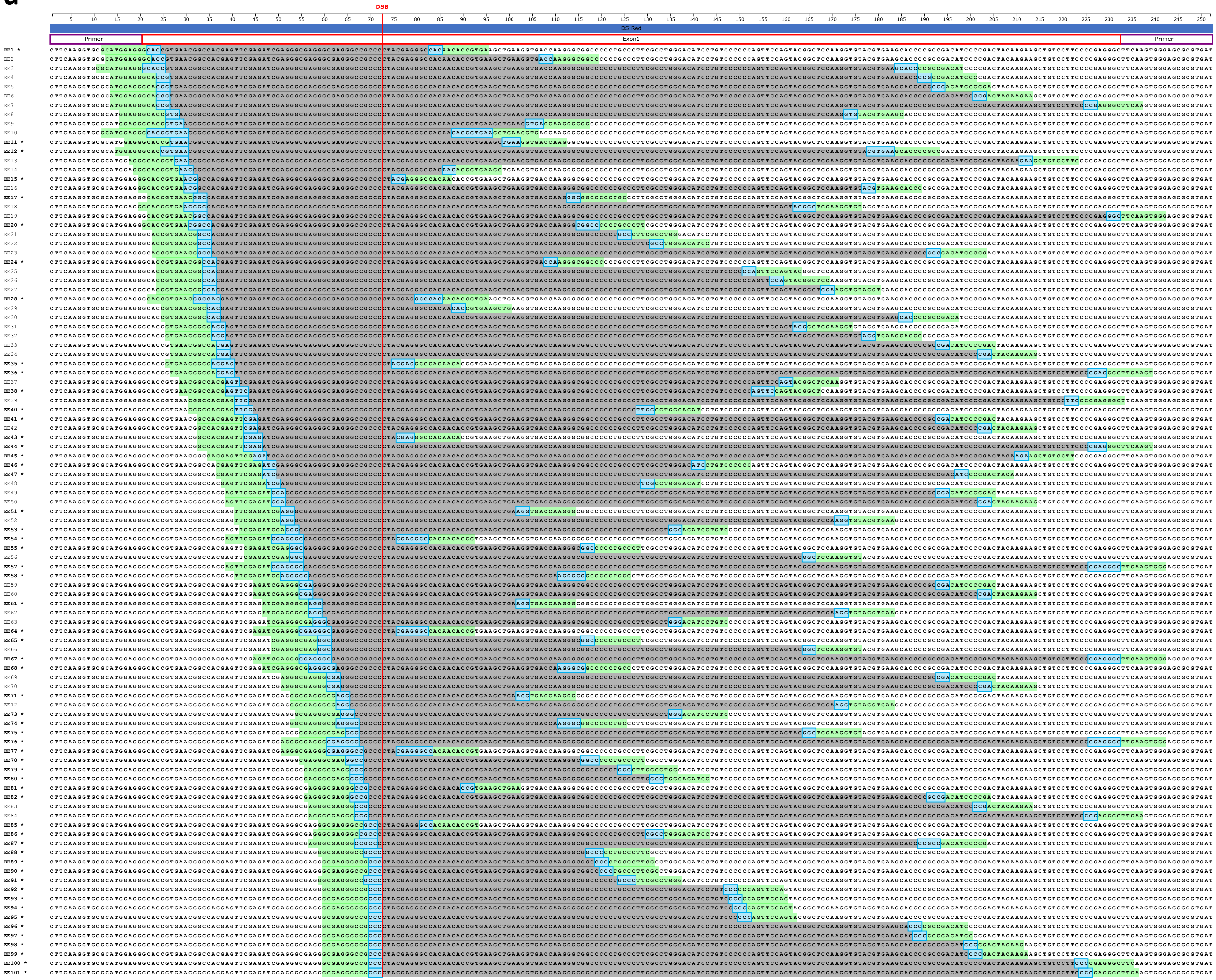

Supplementary Figure 9

e

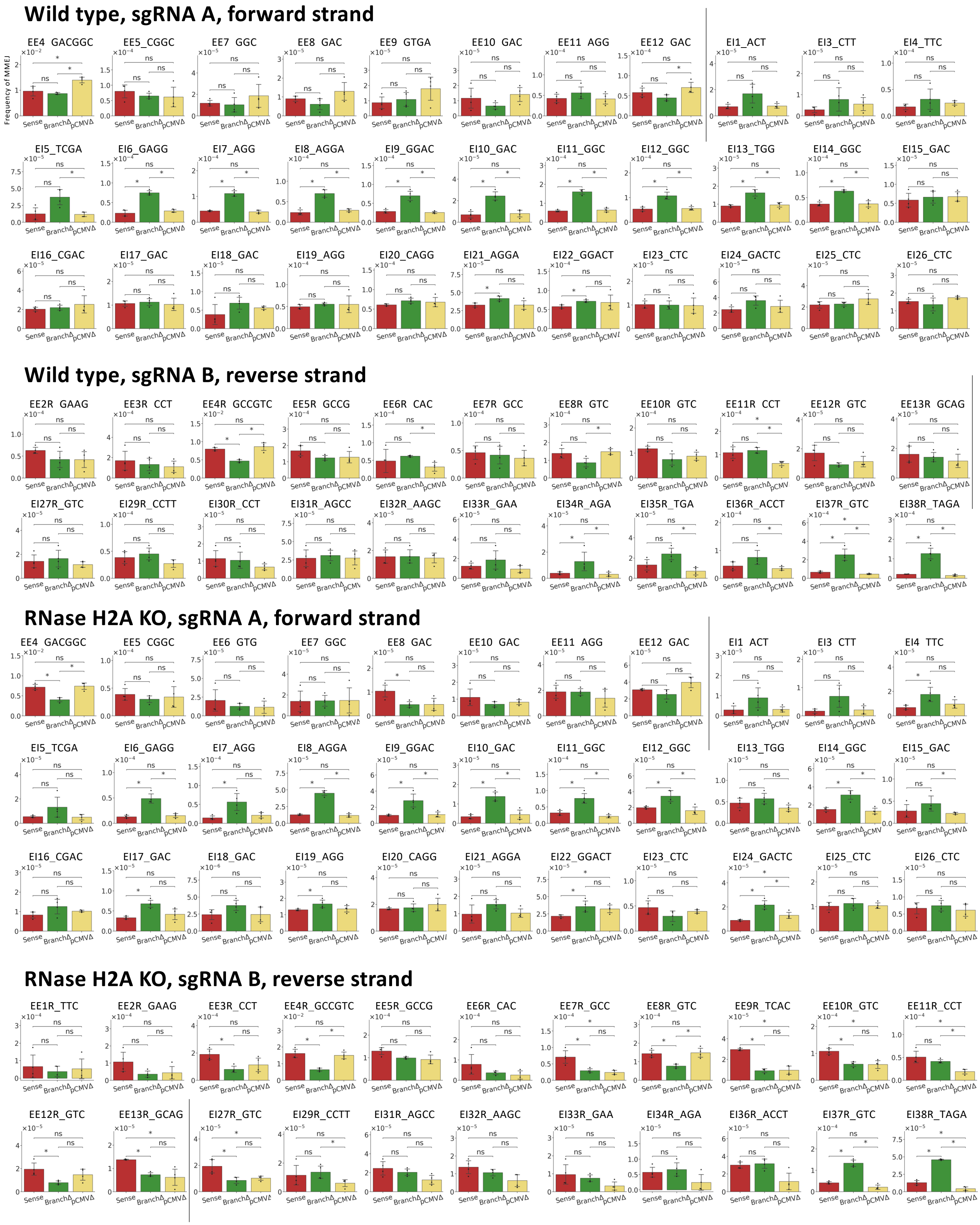

Supplementary Figure 9

f

Wild type, sgRNA E, forward strand

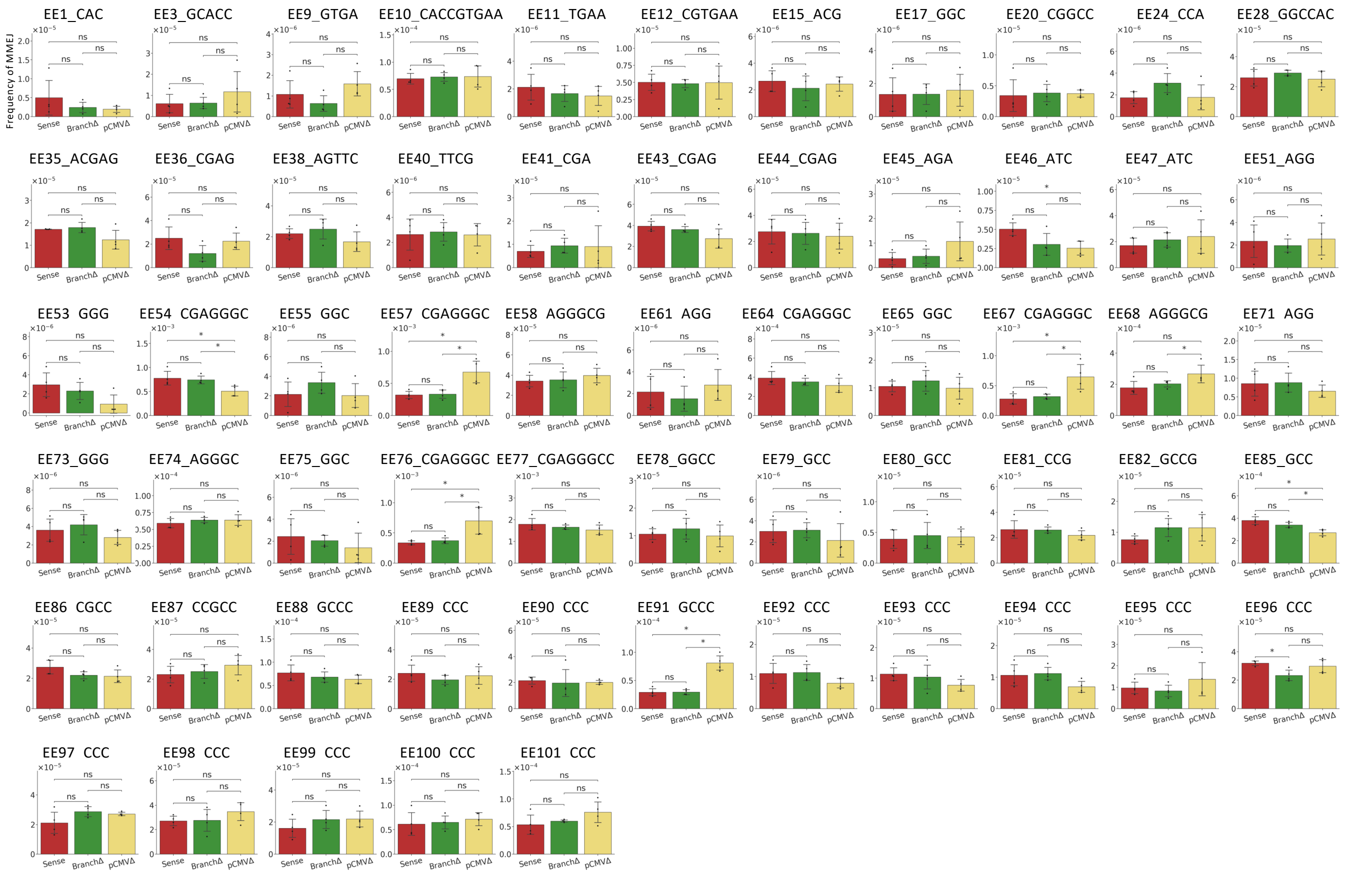

Supplementary Figure 9

g

HEK-293T wild type

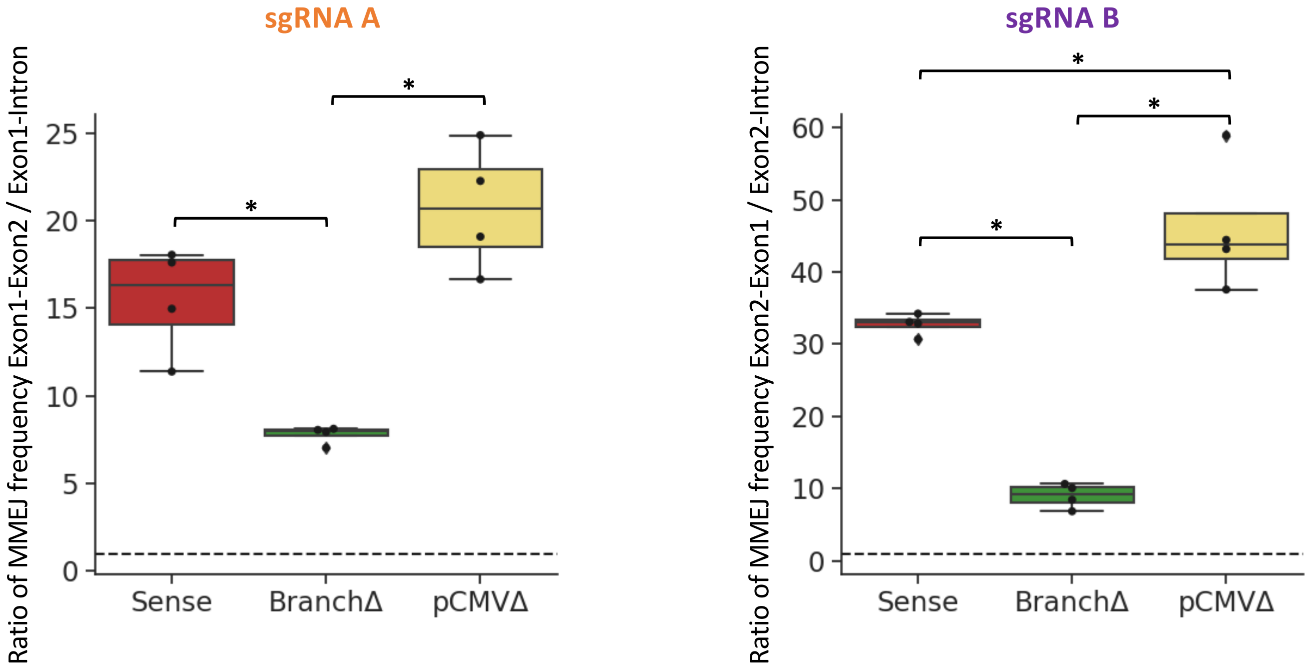

Ratio of MMEJ frequency
[Sum of MMEJ freq using the exon-exon microhomology] / [Sum of MMEJ freq using the exon-intron microhomology]

HEK-293T RNase H2A KO

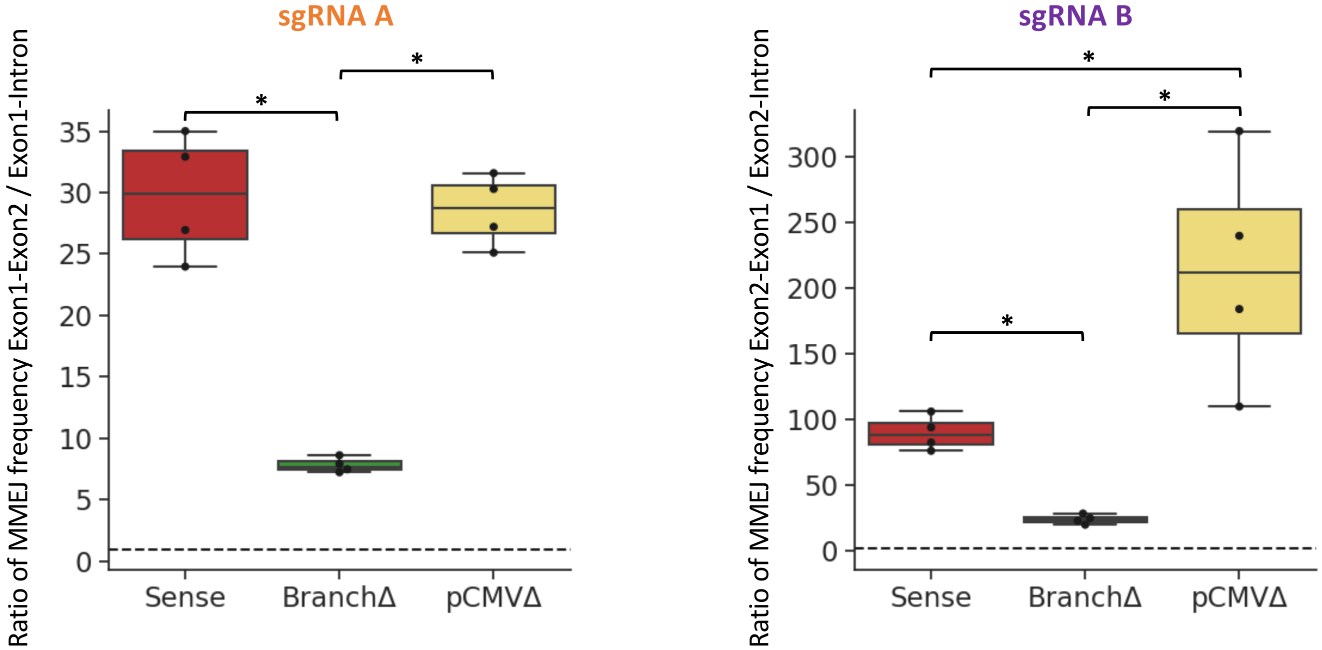

Ratio of MMEJ frequency
[Sum of MMEJ freq using the exon-exon microhomology] / [Sum of MMEJ freq using the exon-intron microhomology]

Supplementary Figure 9 | The spliced transcript facilitates MMEJ between exon-exon microhomologies, while the non-spliced transcript facilitates MMEJ between exon-intron microhomologies.

a, Schemes of regions in which microhomology pairs were identified within the sequenced area to determine the frequency of MMEJ following a DSB by the sgRNA A, B or E. The microhomology pairs were categorized into two groups for a DSB induced by sgRNA A or B: exon-exon and exon-intron. Light blue lines indicate examples of microhomologies; the black-parallel lines show the DSB site; the dark green box shows the region containing the branch site. **b,** Schemes showing all microhomology pairs 3 bp or longer between the two primer sequences in the Sense and pCMVΔ constructs, sgRNA A, forward strand. The *x*-axis shows nucleotide positions from the 5′-primer sequence. First row: *DsRed* gene exons (solid blue), intron (solid green). Second row: primer sequence (purple outline), Exon1/Exon2 (blue outline), Intron (green outline), branch site (dark green outline). DSB position is indicated with a red vertical line. Left margin labels are microhomology IDs (EE, exon-exon; EI, exon-intron); a bold asterisk indicates MMEJ products detected in the sequencing data. Each following row shows the nucleotide sequence of the construct between primer sequences; region deleted by MMEJ repair (gray highlight); microhomology pair (light blue highlight); ± 10-bp flanking region of microhomology pair used for MMEJ detection in the sequencing data (light green highlight). **c,** As in (b) for Sense and pCMVΔ constructs, sgRNA B, reverse strand. **d,** As in (b) for Sense, pCMVΔ, and BranchΔ constructs, sgRNA E, forward strand. **e,** MMEJ frequencies from each microhomology pair detected in the sequencing libraries following a DSB by the sgRNA A or B in the Sense (red), BranchΔ (green), and pCMVΔ (yellow) constructs of wild-type and RNase H2A KO cells. The ID of the microhomology pair being analyzed is shown on top of each bar graph. Plotted data are the mean ± s.d. of the 4 biological replicates with the individual values shown as dots; N=4. The 'ns' on the bar graphs means a non-significant difference (*P*-value > 0.05, two-tailed Mann-Whitney *U* test). **f,** MMEJ frequencies from each microhomology pair detected in the sequencing libraries following a DSB by the sgRNA E. Plotted data are the mean ± s.d. of the 4 biological replicates with the individual values shown as dots; N=4. The 'ns' on the bar graphs means a non-significant difference (*P*-value > 0.05, two-tailed Mann-Whitney *U* test). **g,** Ratio of MMEJ frequencies for the exon-exon and exon-intron following a DSB by the sgRNA A or B in the Sense (red), BranchΔ (green), and pCMVΔ (yellow) constructs of wild-type (top) and RNase H2A KO (bottom) cells. The ratio was calculated by dividing the sum of MMEJ frequencies from the exon-exon microhomology pairs by the sum of MMEJ frequencies from the exon-intron microhomology pairs in each sequencing library, N=4. The median of the points is shown as the middle line of the box. The first and third quartiles are indicated by the box frames and the whiskers represent the largest point not more than 1.5 interquartile range (IQR) beyond the box frame. All data points outside the whiskers are classified as outliers and shown as diamond points. *, *P* = 0.029 (two-tailed Mann-Whitney *U* test). Source data are provided as a Source Data file.

Supplementary Figure 10

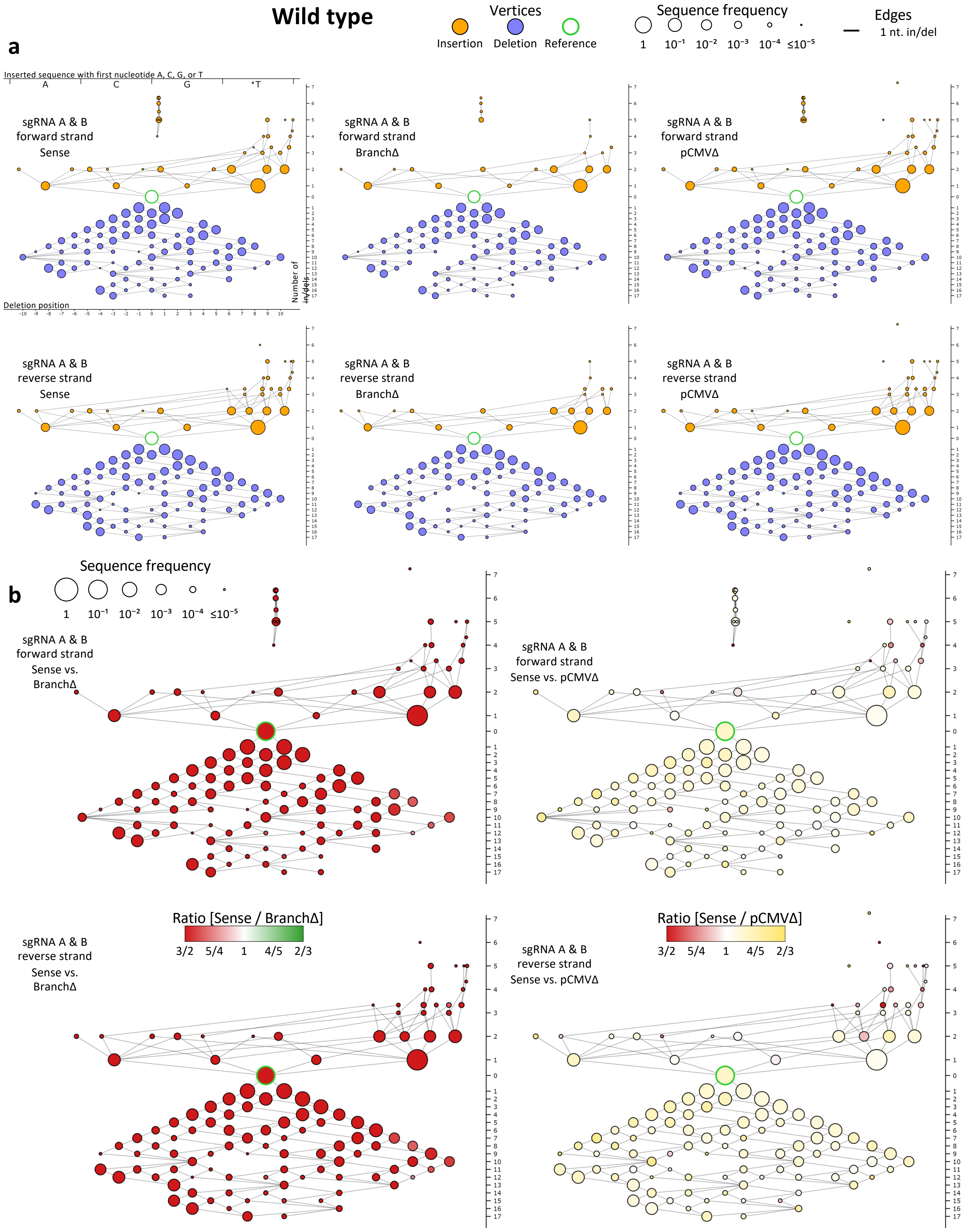

Supplementary Figure 10

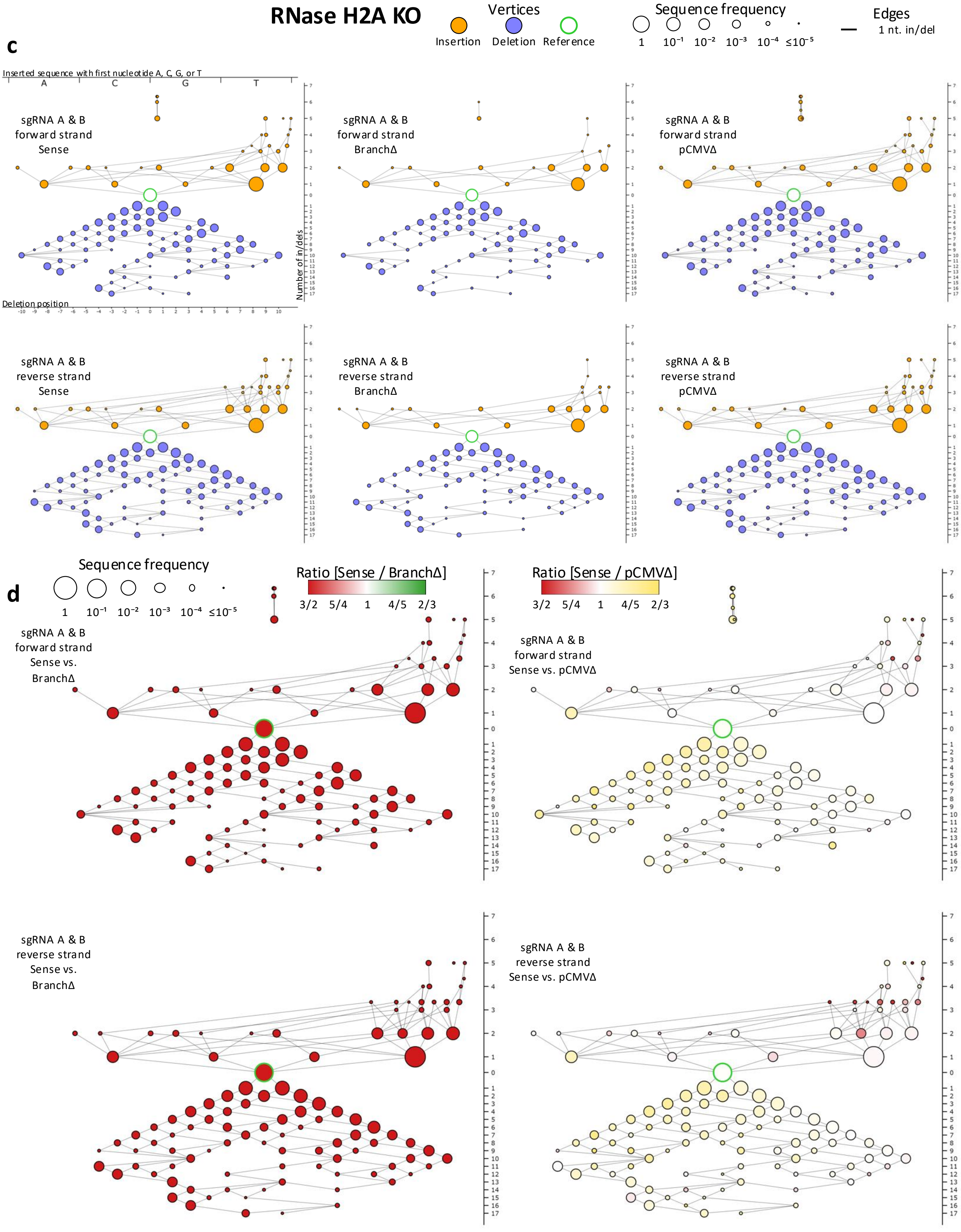

Supplementary Figure 10

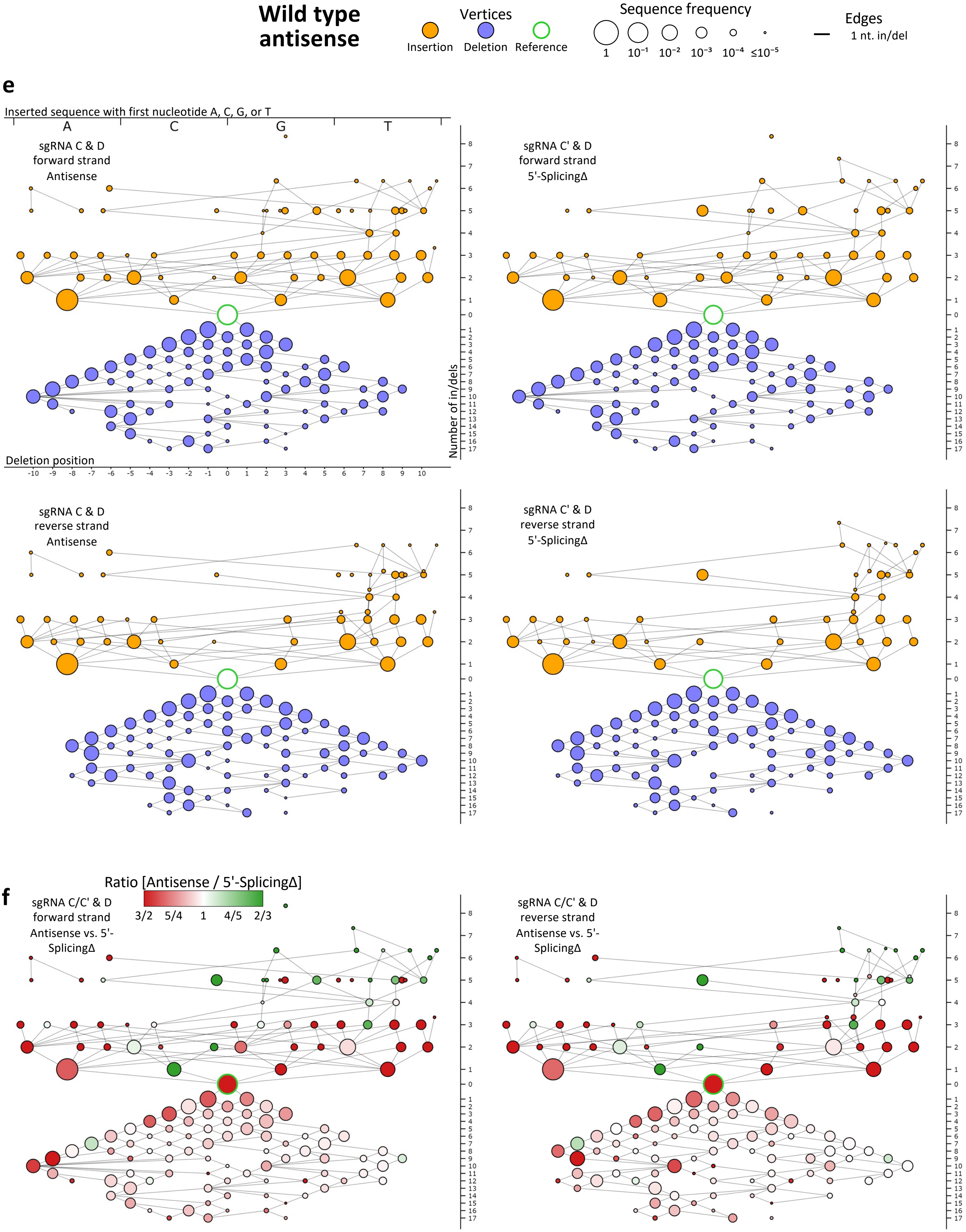

Supplementary Figure 10 | Transcript RNA promotes double-strand gap repair in a sequence-dependent manner via NHEJ – all data for the sense and antisense constructs.

a, Individual variation-distance graphs illustrating sequence variations within DSB-sequence windows observed after gap induction by sgRNA A and sgRNA B in the Sense, BranchΔ, and pCMVΔ constructs of wild-type cells. Data obtained by sequencing the forward (top) and the reverse (bottom) strands. **b,** Comparison variation-distance graphs of the DSB-sequence windows obtained after gap induction by sgRNA A and sgRNA B for the Sense vs. the BranchΔ construct (left) or for the Sense vs. the pCMVΔ construct (right) of wild-type cells. The vertices represent the same DSB-sequence windows as for the individual graphs while the vertex colors specify the relative frequency in the Sense (red) vs. the BranchΔ (green) construct, or the Sense (red) vs. the pCMVΔ (yellow) construct; the vertex sizes show the log of the maximum of the two mean frequencies of the corresponding DSB-sequence window in the two compared constructs. Data obtained by sequencing the forward (top) and the reverse (bottom) strands. **c,** Same as in (a) for constructs of RNase H2A KO cells. **d,** Same as in (b) for constructs of RNase H2A KO cells. **e,** Individual variation-distance graphs illustrating sequence variations within DSB-sequence windows observed after gap induction by sgRNA C/C' and sgRNA D in the Antisense and 5′-SplicingΔ constructs of wild-type cells. Data obtained by sequencing the forward (top) and the reverse (bottom) strands. **f,** Comparison variation-distance graphs of the DSB-sequence windows obtained after gap induction by sgRNA C/C' and sgRNA D for the Antisense vs. the 5′-SplicingΔ construct of wild-type cells. The vertices represent the same DSB-sequence windows as for the individual graphs while the vertex colors specify the relative frequency for the Antisense (red) vs. the 5′-Splicing (green) construct; the vertex sizes show the log of the maximum of the two mean frequencies of the corresponding DSB-sequence window in the two compared constructs. Data obtained by sequencing the forward (left) and the reverse (right) strands. Refer to **Supplementary Figure 7** for the description of variation-distance graphs. For graphs showing reverse-strand data, the sequences are reverse-complemented prior to computing *xy*-coordinates so that they correspond to forward-strand sequence coordinates.

Supplementary Figure 11

a

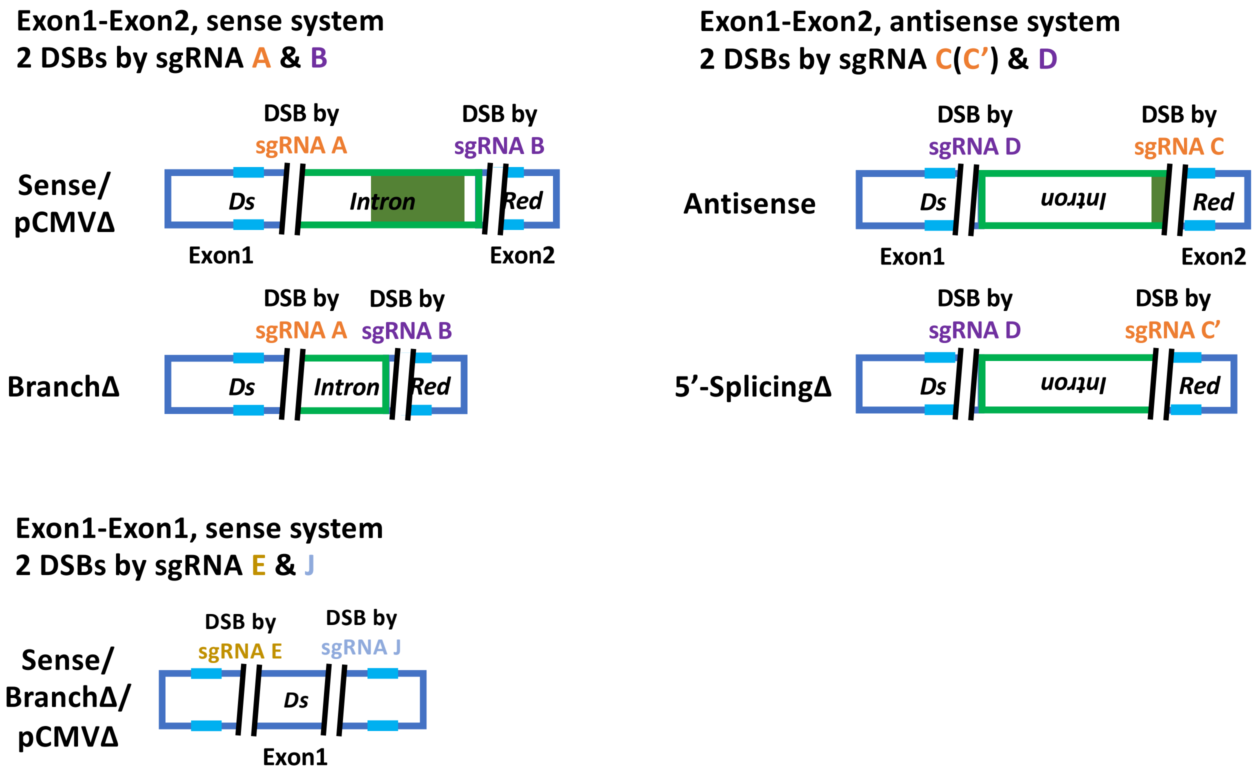

Supplementary Figure 11

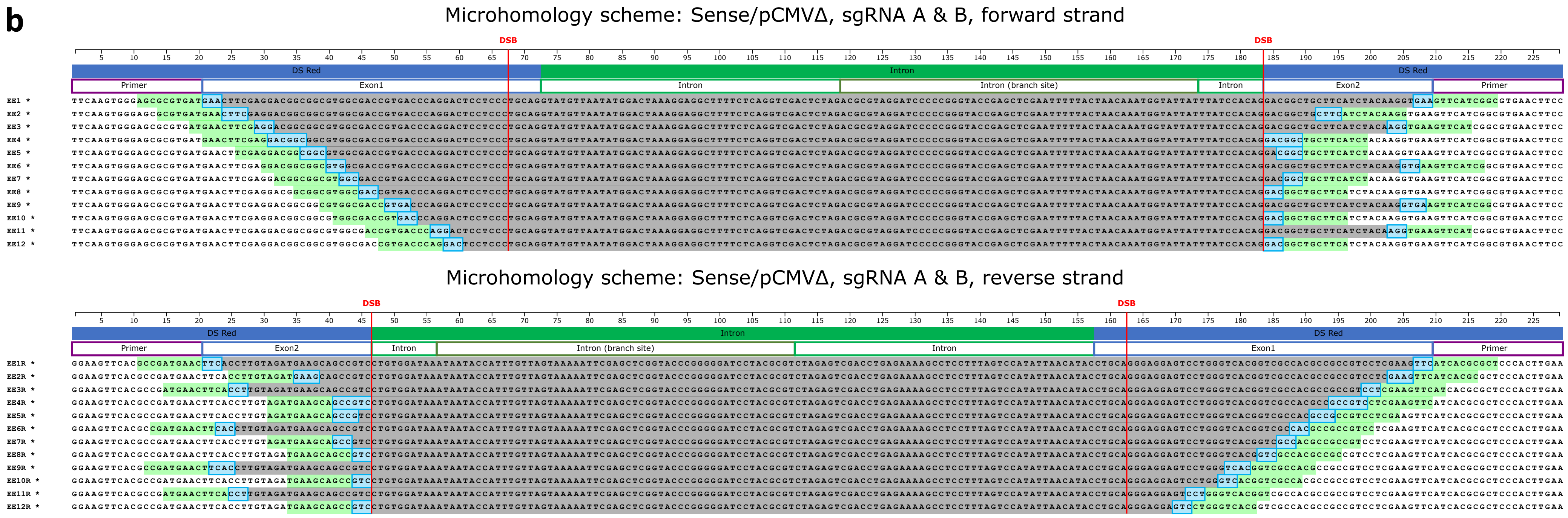

Supplementary Figure 11

c

Microhomology scheme: Sense/pCMVΔ/BranchΔ, sgRNA E & J, forward strand

Microhomology scheme: Sense/pCMVΔ/BranchΔ, sgRNA E & J, reverse strand

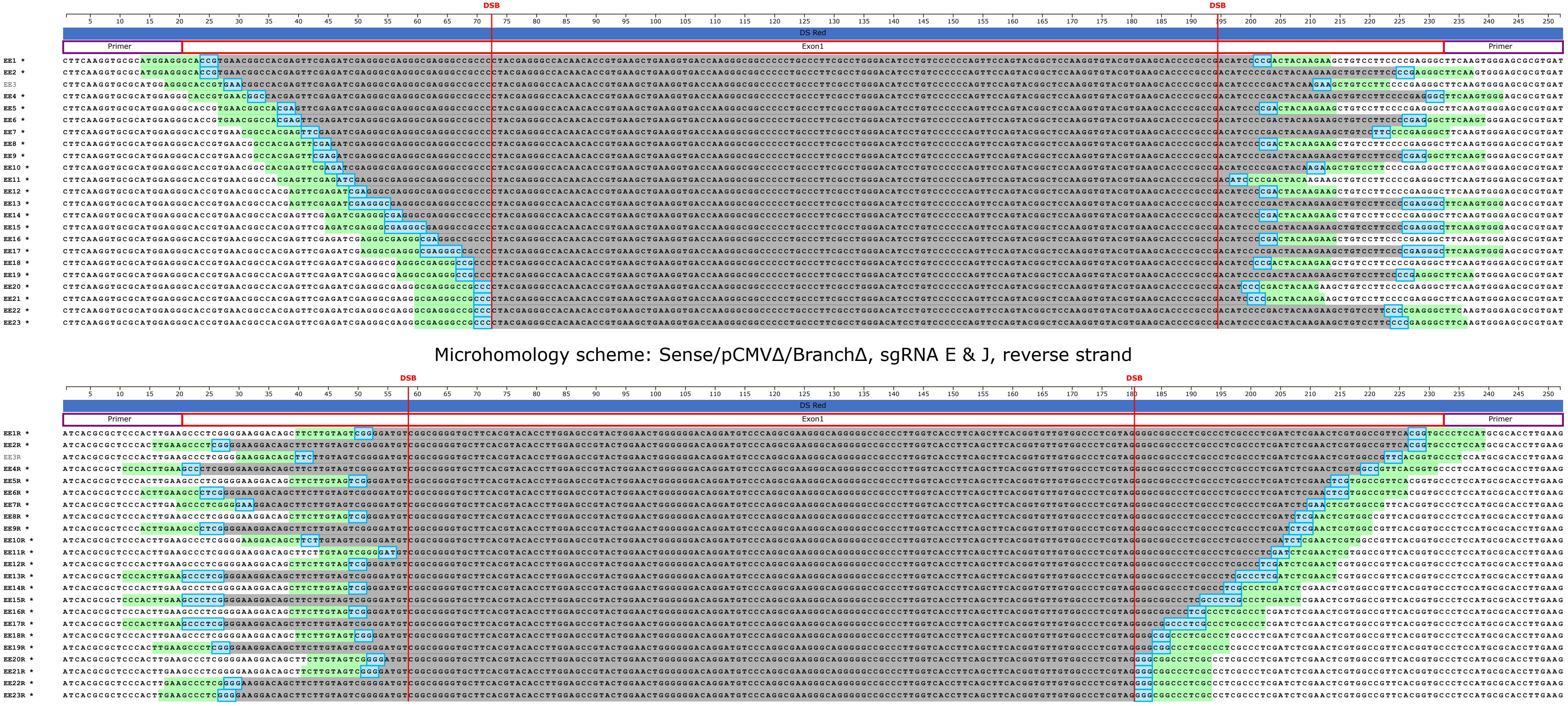

Supplementary Figure 11

d

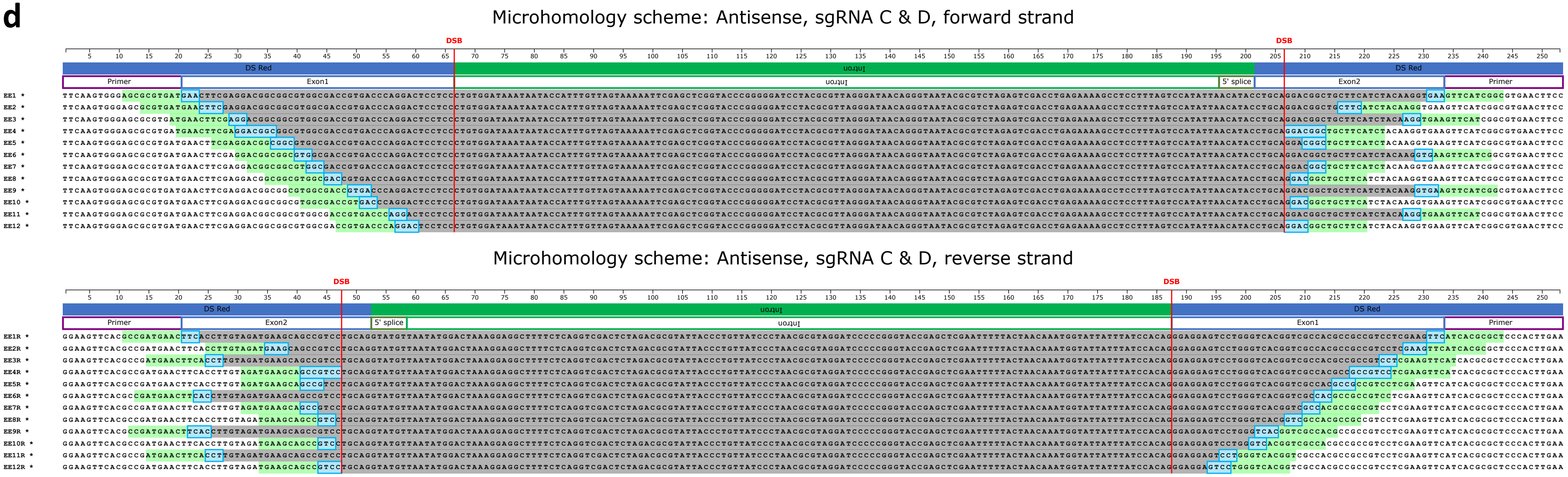

Supplementary Figure 11

e

Wild type, sgRNA A and B (2 DSBs), forward strand

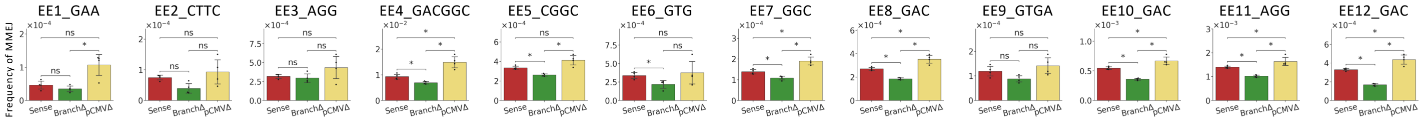

Wild type, sgRNA A and B (2 DSBs), reverse strand

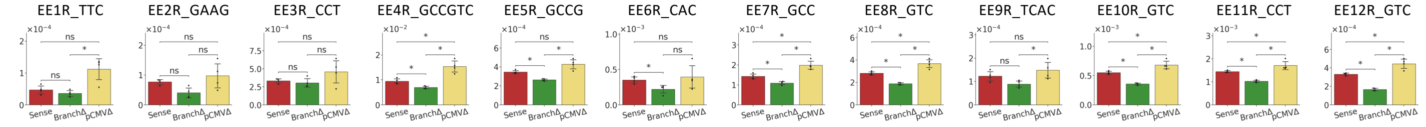

RNase H2A KO, sgRNA A and B (2 DSBs), forward strand

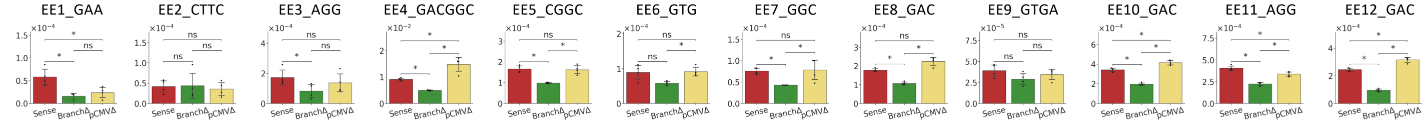

RNase H2A KO, sgRNA A and B (2 DSBs), reverse strand

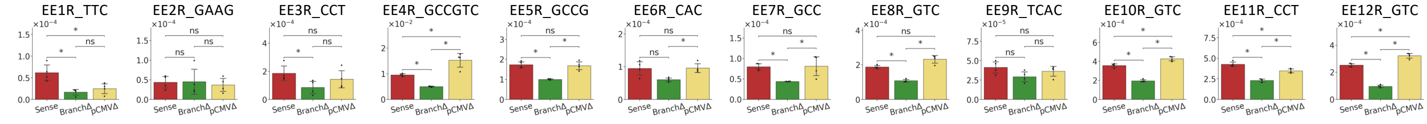

f

Wild type, sgRNA E and J (2 DSBs), forward strand

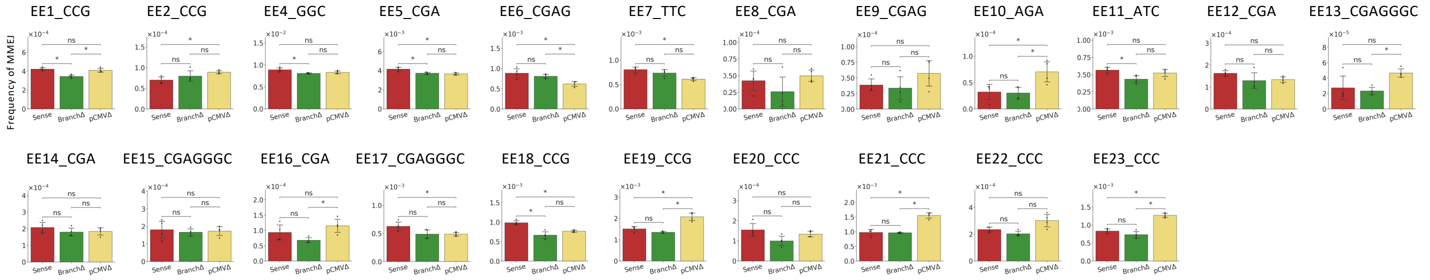

Wild type, sgRNA E and J (2 DSBs), reverse strand

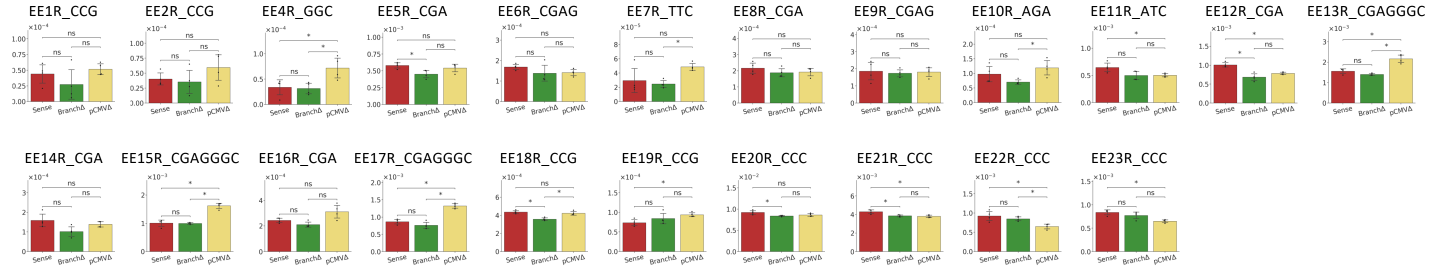

Supplementary Figure 11

g

Antisense, wild type, sgRNA C (or C’) and D (2 DSBs), forward strand

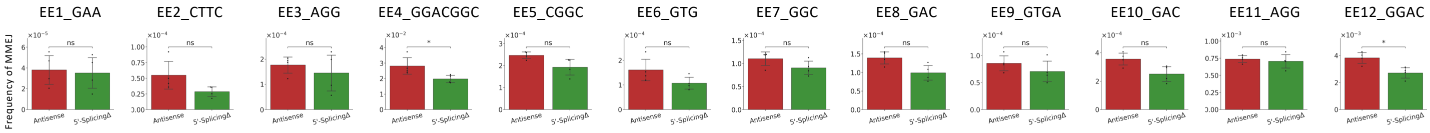

Antisense, wild type, sgRNA C (or C’) and D (2 DSBs), reverse strand

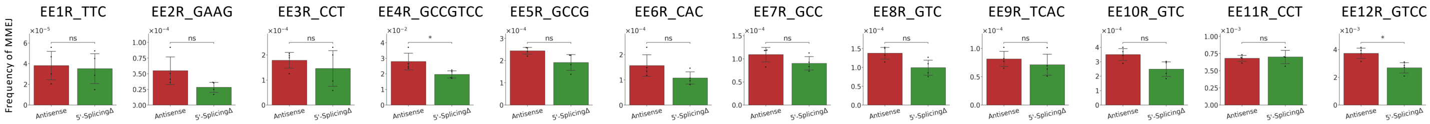

Supplementary Figure 11 | Transcript RNA promotes double-strand gap repair in a sequence-dependent manner via MMEJ – all data for the sense and antisense constructs.

a, Schemes of regions in which Exon1-Exon2 microhomology pairs were identified within the sequenced area to determine the frequency of MMEJ following a gap by the sgRNAs A and B in the sense constructs (left), or by the sgRNAs C/C' and D in the antisense constructs (right), or Exon1-Exon1 microhomology pairs identified following a gap by the sgRNAs E and J in the sense constructs (bottom). Light blue lines indicate examples of microhomologies; the black, parallel lines show the DSB sites; the dark, green box shows the region containing the branch site for the Sense and pCMVΔ constructs, and the region with the 5′-splice site for the Antisense construct. **b,** Schemes showing all microhomology pairs 3 bp or longer between the two primer sequences in the Sense and pCMVΔ constructs, sgRNAs A and B, forward strand (top), reverse strand (bottom). The *x*-axis shows nucleotide positions from the 5′-primer sequence. First row: *DsRed* gene exons (solid blue), Intron (solid green). Second row: primer sequence (purple outline), Exon1/Exon2 (blue outline), intron (green outline), branch site (dark green outline). DSB positions are indicated with two red vertical lines. Left margin labels are microhomology IDs (EE, exon-exon); bold/asterisk indicates MMEJ products detected in the sequencing data. Each following row shows the nucleotide sequence of the construct between primer sequences; region deleted by MMEJ repair (gray highlight); microhomology pair (light blue highlight); ± 10-bp flanking region of microhomology pair used for MMEJ detection in the sequencing data (light green highlight). **c,** As in (b) for sense constructs Sense, pCMVΔ, and BranchΔ, sgRNAs E and J, forward strand (top), reverse strand (bottom). **d,** As in (b) for Antisense construct, sgRNAs C and D, forward strand (top), reverse strand (bottom). The reversed intron in the antisense constructs is indicated with an upside-down label; 5′-splice site (dark green outline). **e, f,** Mean of frequencies of MMEJ repair from each microhomology pair detected in the sequencing libraries following a gap by the sgRNAs A and B (e) or sgRNAs E and J (f) in the Sense (red), BranchΔ (green), and pCMVΔ (yellow) constructs of wild-type and RNase H2A KO cells, and **g,** following a gap by the sgRNAs C/C' and D in the Antisense (red) and 5′-SplicingΔ (green) constructs of wild-type cells. Plotted data are the mean ± s.d. of the 4 biological replicates with the individual values shown as dots. The ID of the microhomology pair being analyzed is on top of each bar graph; N=4. The 'ns' on the bar graphs means a non-significant difference (P-value > 0.05, two-tailed Mann-Whitney U test). *, P = 0.029 (two-tailed Mann-Whitney U test). Source data are provided as a Source Data file.

Supplementary Figure 12

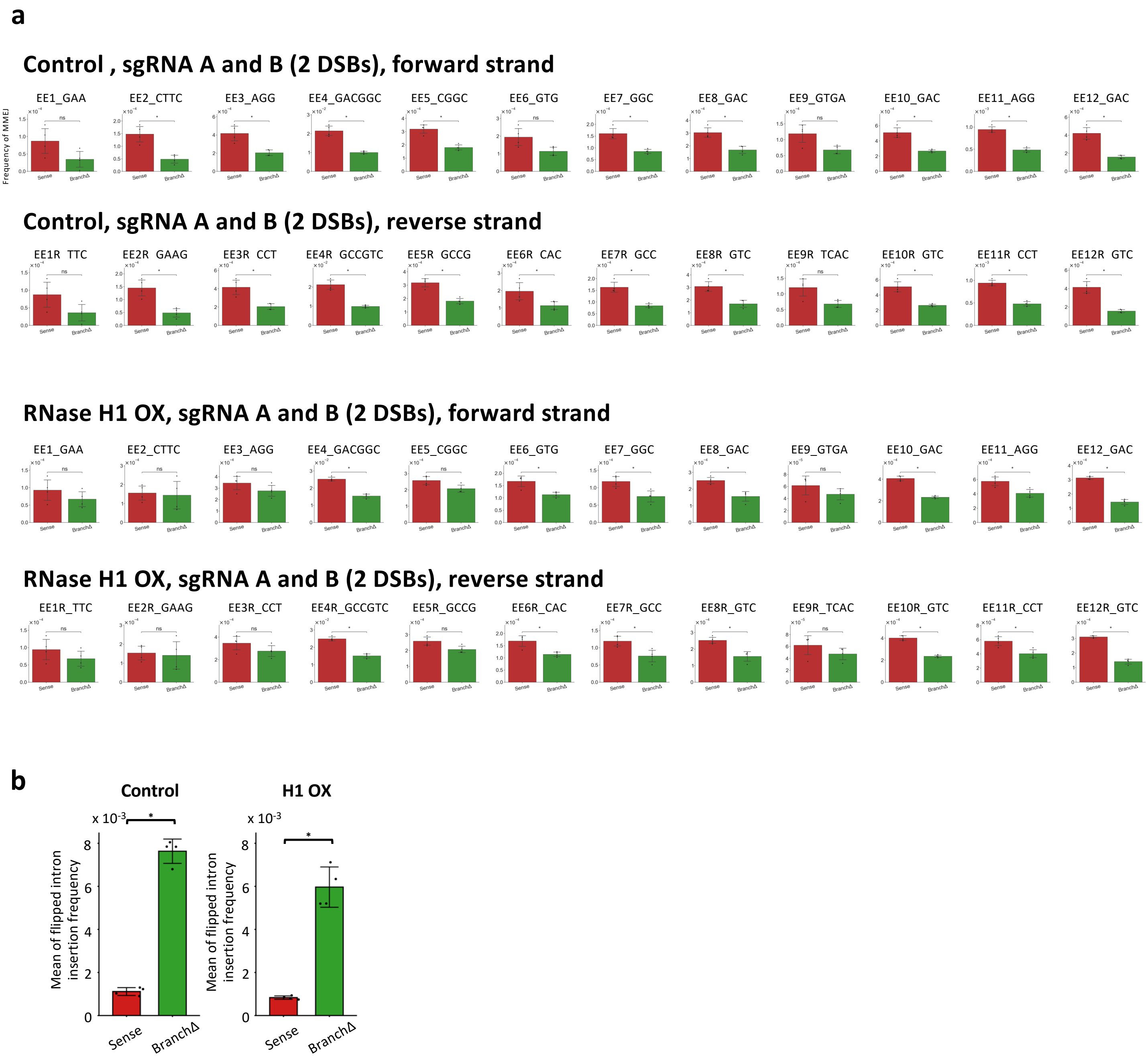

Supplementary Figure 12 | Impact of transcript RNA on MMEJ and flipped intron repair following overexpression of RNase H1.

a, Mean of frequencies of MMEJ repair from each microhomology pair detected in the sequencing libraries following a gap by the sgRNAs A and B in the Sense (red) and BranchΔ (green) constructs of Control and H1 OX. Plotted data are the mean ± s.d. of the 4 biological replicates with the individual values shown as dots. The ID of the microhomology pair being analyzed is on top of each bar graph; N=4. The 'ns' on the bar graphs means a non-significant difference (P-value > 0.05, two-tailed Mann-Whitney U test). *, P = 0.029 (two-tailed Mann-Whitney U test). **b,** Frequency of intron flipping caused by re-capture of the intron via NHEJ following a gap by the sgRNAs A and B in the Sense (red) and BranchΔ (green) constructs of Control and H1 OX. Plotted data are the mean ± s.d. of the 4 biological replicates with the individual values shown as dots; N=4. *, P = 0.029 (two-tailed Mann-Whitney U test). Source data are provided as a Source Data file.

Supplementary Figure 13

HEK293 WT, sgRNA A, Individual Exon-Exon MMEJ

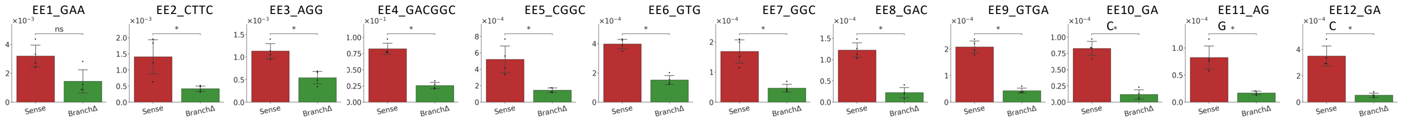

HEK293T WT, sgRNA A, Individual Exon-Exon MMEJ

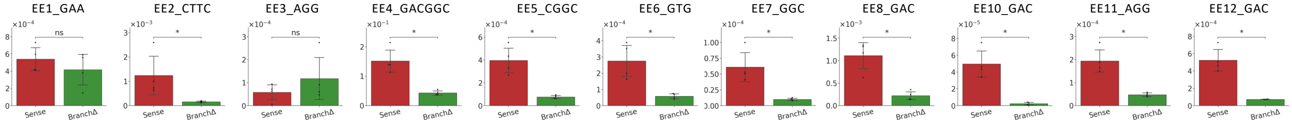

HEK293 WT, sgRNA A, Individual Exon-Intron MMEJ

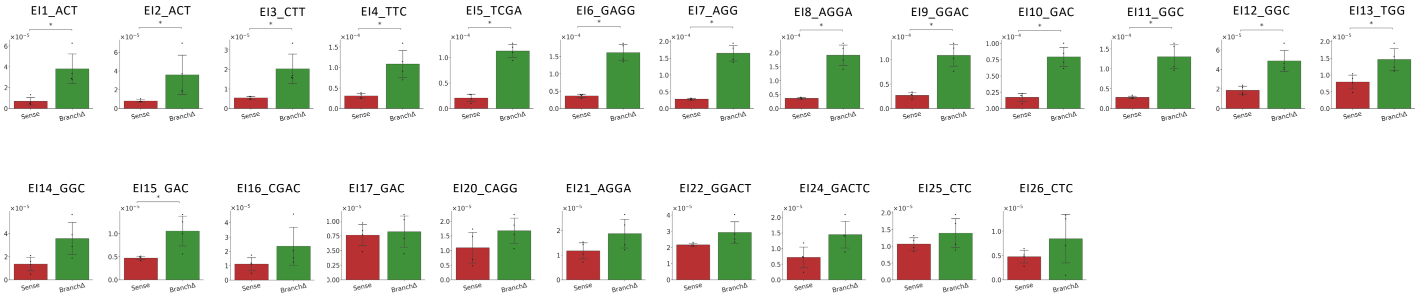

HEK293T WT, sgRNA A, Individual Exon-Intron MMEJ

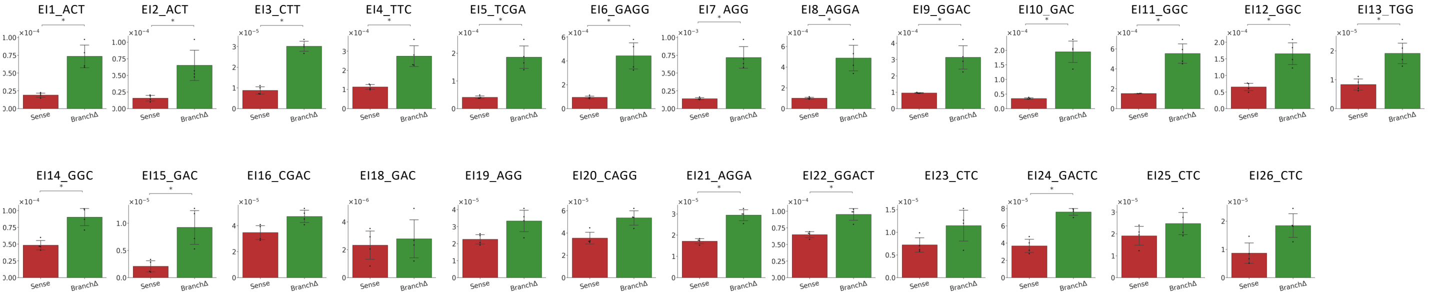

HEK293 WT, 2DSB, Individual Exon-Exon MMEJ

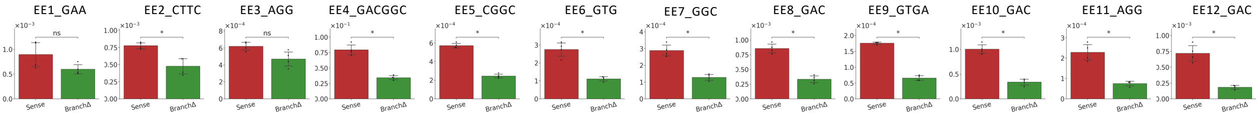

Supplementary Figure 13 | RNA-mediated DNA double-strand break/gap repair by MMEJ is independent of DNA replication.

MMEJ frequencies from each microhomology pair detected in the sequencing libraries following a DSB by the sgRNA A or double-strand gap by the sgRNAs A and B in the Sense (red) and BranchΔ (green) constructs of HEK293 and HEK293T cells. The ID of the microhomology pair being analyzed is shown on top of each bar graph. Plotted data are the mean ± s.d. of the 4 biological replicates with the individual values shown as dots; N=4. The 'ns' on the bar graphs means a non-significant difference (P-value > 0.05, two-tailed Mann-Whitney U test). Source data are provided as a Source Data file.

Supplementary Figure 14

Antisense

Forward sequencing primer

DSB by sgRNA C

GCCTCTTTAAAAGCTTGACCGAGAGCAATCCCGCAGTCTTCAGTGTGGTGATGGTCGTCTATGTGTAAGTCACCAATGCACTCAACGATTAGCGACCAGCCGGAATGCTT
CGGAGAAATTTTCGAACTGGCTCTCGTTAGGGCGTCAGAAGTCACCACACTACCAGCAGATACACATTCAGTGGTTACGTGAGTTGCTAATCGCTGGTCGGCCTTACGAA

ɛs

PAM

Intron

GGGTATGTTAATATGGACTAAAGGAGGCTTTTCTGCAGGTCGACTCTAGAACCACTCTACAAAACCAAAACCAGGGTTTATAAAATTATACTGTTGCGGAAAGCTGAAAC
CCCATACAATTATACCTGATTTCCTCCGAAAAGACGTCCAGCTGAGATCTTGGTGAGATGTTTTGGTTTTGGTCCCAAATATTTTAATATGACAACGCCTTTCGACTTTG

sgRNA C

Sequence with branch site

TAAAAGAAAAACCCGACTATGCTATTTTAATCATTGAAAACGAATTTATTTAGATCCCCGTACAGGATCCCCCGGGTACCGAGCTCGAATTTTTACTAACAAATGGTATT
ATTTTCTTTTTGGGCTGATACGATAAAATTAGTAACTTTTGCTTAAATAAATCTAGGGGCATGTCCTAGGGGGCCCATGGCTCGAGCTTAAAAATGATTGTTTACCATAA

DSB by sgRNA D

ATTTCCAACAGCCAGAGCATGTATCATATGGTCCAGAAACCCTATACCTGTGTGGACGTTAATCACTTGCGATTGTGTGGCCTGTTCTGCTACTG 3′
TAAAGGTTGTCGGTCTCGTACATAGTATACCAGGTCTTTGGGATATGGACACACCTGCAATTAGTGAACGCTAACACACCGGACAAGACGATGAC 5′

PAM sgRNA D

!ɥ

BranchΔ

Forward sequencing primer

DSB by sgRNA C

GCCTCTTTAAAAGCTTGACCGAGAGCAATCCCGCAGTCTTCAGTGTGGTGATGGTCGTCTATGTGTAAGTCACCAATGCACTCAACGATTAGCGACCAGCCGGAATGCTT
CGGAGAAATTTTCGAACTGGCTCTCGTTAGGGCGTCAGAAGTCACCACACTACCAGCAGATACACATTCAGTGGTTACGTGAGTTGCTAATCGCTGGTCGGCCTTACGAA

ɛs

PAM

Intron

GGGTATGTTAATATGGACTAAAGGAGGCTTTTCTGCAGGTCGACTCTAGAACCACTCTACAAAACCAAAACCAGGGTTTATAAAATTATACTGTTGCGGAAAGCTGAAAC
CCCATACAATTATACCTGATTTCCTCCGAAAAGACGTCCAGCTGAGATCTTGGTGAGATGTTTTGGTTTTGGTCCCAAATATTTTAATATGACAACGCCTTTCGACTTTG

sgRNA C

DSB by sgRNA D

TAAAAGAAAAACCCGACTATGCTATTTTAATCATTGAAAACGAATTTATTTAGATCCCCGTACAGGAAAATGGTATTATTTCCAACAGCCAGAGCATGTATCATATGGTC
ATTTTCTTTTTGGGCTGATACGATAAAATTAGTAACTTTTGCTTAAATAAATCTAGGGGCATGTCCTTTTACCATAATAAAGGTTGTCGGTCTCGTACATAGTATACCAG

PAM sgRNA D

CAGAAACCCTATACCTGTGTGGACGTTAATCACTTGCGATTGTGTGGCCTGTTCTGCTACTG 3′
GTCTTTGGGATATGGACACACCTGCAATTAGTGAACGCTAACACACCGGACAAGACGATGAC 5′

!ɥ

Reverse sequencing primer

Supplementary Figure 14 | DNA sequence of the *his3* loci of the yeast constructs.
Blue sequence, exons of the *his3* gene; green sequence, intron; underlined green sequence, canonical GT- and -AG splice sites of the intron; underlined dark green sequence, intron sequence containing the branch site. Black arrows, sequencing primers; yellow highlighted sequence, PAM site; orange highlighted sequence, sequence of sgRNA C; purple highlighted sequence, sequence of sgRNA D. Vertical black bar, site of DSB by sgRNA.

Supplementary Figure 15

a

Construct	spliced transcript frequency	non-spliced transcript frequency	alt-spliced transcript frequency	non-canonical alt-spliced frequency	unaligned frequency
WT, antisense	**0.23**	0.73	0	0.029	0.0066
WT, branchΔ	0.00057	**0.99**	0	0.0020	0.0058
spt3, antisense	**0.16**	0.82	0	0.0160	0.0020
spt3, branchΔ	0.0001	**0.9981**	0	0.0001	0.0017
spt3 rnh1 rnh201, antisense	**0.21**	0.76	0	0.0258	0.0028
spt3 rnh1 rnh201, branchΔ	0.0001	**0.9969**	0	0.0006	0.0023

b

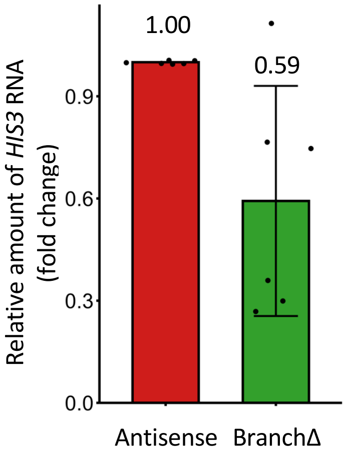

c

Construct	spliced transcript frequency	non-spliced transcript frequency	alt-spliced transcript frequency	non-canonical alt-spliced frequency	unaligned frequency
WT, antisense	**0.077**	0.91	0	0.0096	0.0013
WT, branchΔ	0.0007	**0.998**	0	0.0003	0.0012
ku70, antisense	**0.15**	0.82	0	0.028	0.0070
ku70, branchΔ	0.0007	**0.98**	0	0.011	0.0061

Supplementary Figure 15 | The RNA transcribed from the BranchΔ yeast construct has no intron splicing in all genomic backgrounds used in this study.

a, Table of RNA-sequencing results of *his3* transcripts from the Antisense and BranchΔ in wild-type*, spt3*, *spt3 rnh1 rnh201* KO cells. RNA-sequencing reads for each transcript of the different constructs were categorized after alignment to the corresponding DNA sequence (details in Methods). Spliced transcript, a transcript in which the intron was spliced out from the canonical GT---AG-splice sites; alt-spliced transcript, a transcript in which splicing happened at alternative GT---AG sites; non-canonical alternative splicing, a transcript sequence that had splicing at sites different from the GT---AG sites. Splicing frequencies were calculated by dividing the number of the spliced-transcript reads by the total number of reads within each RNA-sequencing library, N=1. **b,** Results of RT-qPCR of the *his3* transcripts generated from each construct. The bar shows the relative amount of the *his3* transcripts from the BranchΔ (green bar) construct compared to the amount of the Sense (red bar) transcripts. Plotted data are the mean fold change ± s.d. of the 6 biological replicates with the individual values shown as dots; N=6. The mean value is shown above each bar. **c,** Table of RNA-sequencing results of *his3* transcripts from the Antisense and BranchΔ in wild-type and *ku70* KO cells, N=1. Source data are provided as a Source Data file.

Supplementary Figure 16

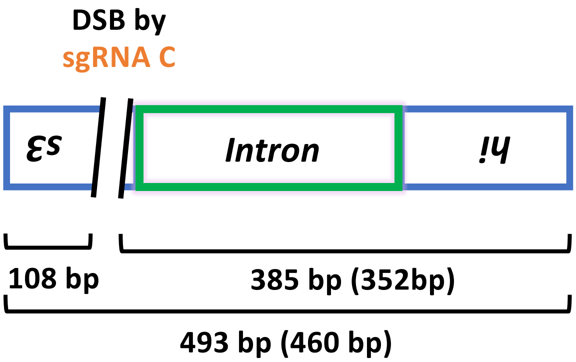

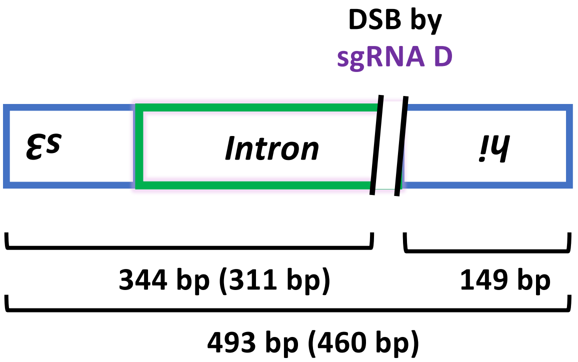

$$DSB\ efficiency = \frac{\frac{108bp + 385bp}{2}}{\frac{108bp + 385bp}{2} + 493bp}$$

(Calculate by molarity)

$$DSB\ efficiency = \frac{\frac{344bp + 149bp}{2}}{\frac{344bp + 149bp}{2} + 493bp}$$

(Calculate by molarity)

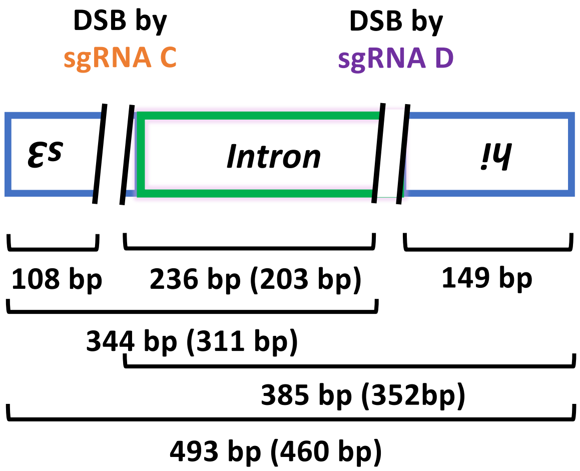

$$2\text{-}DSB\ efficiency = \frac{236bp}{344bp + 385bp + 236bp + 493bp}$$

(Calculate by molarity)

**sgRNA C**	1st	2nd	3rd	4th	Average
Antisense	**0.945**	**0.938**	**0.931**	**0.929**	**0.936**
BranchΔ	**0.922**	**0.932**	**0.938**	**0.947**	**0.934**

**sgRNA D**	1st	2nd	3rd	4th	5th	Average
Antisense	**0.913**	**0.939**	**0.944**	**0.949**	**0.934**	**0.936**
BranchΔ	**0.932**	**0.924**	**0.950**	**0.930**	**0.943**	**0.936**

**2-DSB**	1st	2nd	3rd	4th	5th	Avg.
Antisense	**0.891**	**0.894**	**0.885**	**0.890**	**0.891**	**0.890**
BranchΔ	**0.885**	**0.886**	**0.895**	**0.890**	**0.891**	**0.890**

Supplementary Figure 16 | The yeast construct with splicing has similar cleavage efficiency by Cas9 to that without splicing.

Scheme of PCR fragments for the yeast Antisense and BranchΔ used for the *in vitro*-cleavage assay. Cas9 cleavage was done using sgRNA C, D or C and D for the Antisense and BranchΔ constructs. The formulas used to calculate the DSB efficiencies from the molarity of each DNA fragment detected by the Bioanalyzer following cleavage by Cas9 with sgRNA C, D, or C and D are shown. The bp sizes in the formulas are those for the Antisense and BranchΔ (in parenthesis when different). Results of the *in vitro*-cleavage assay are shown in the tables underneath the construct schemes.

Supplementary Figure 17

Wild type

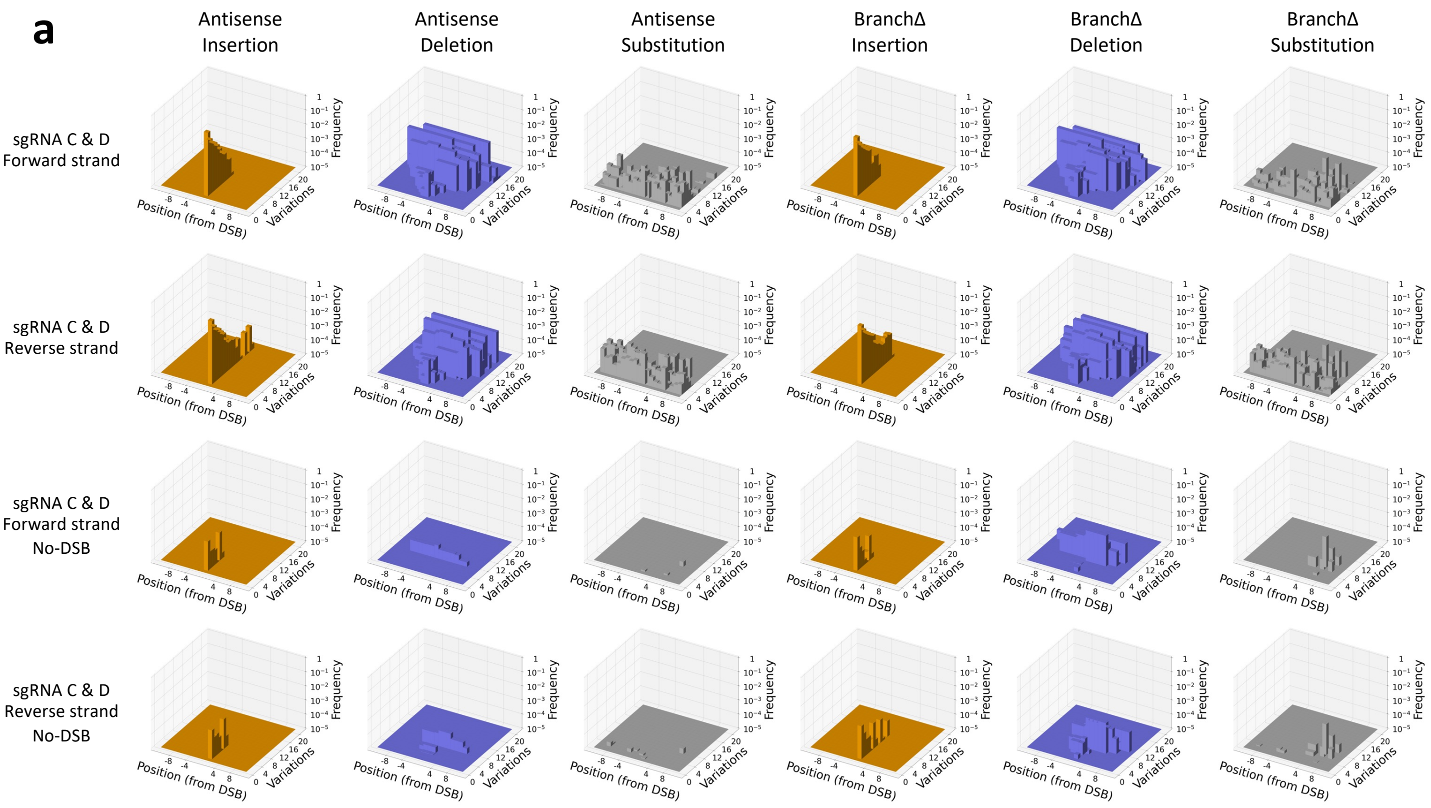

Supplementary Figure 17

Wild type (*spt3*)

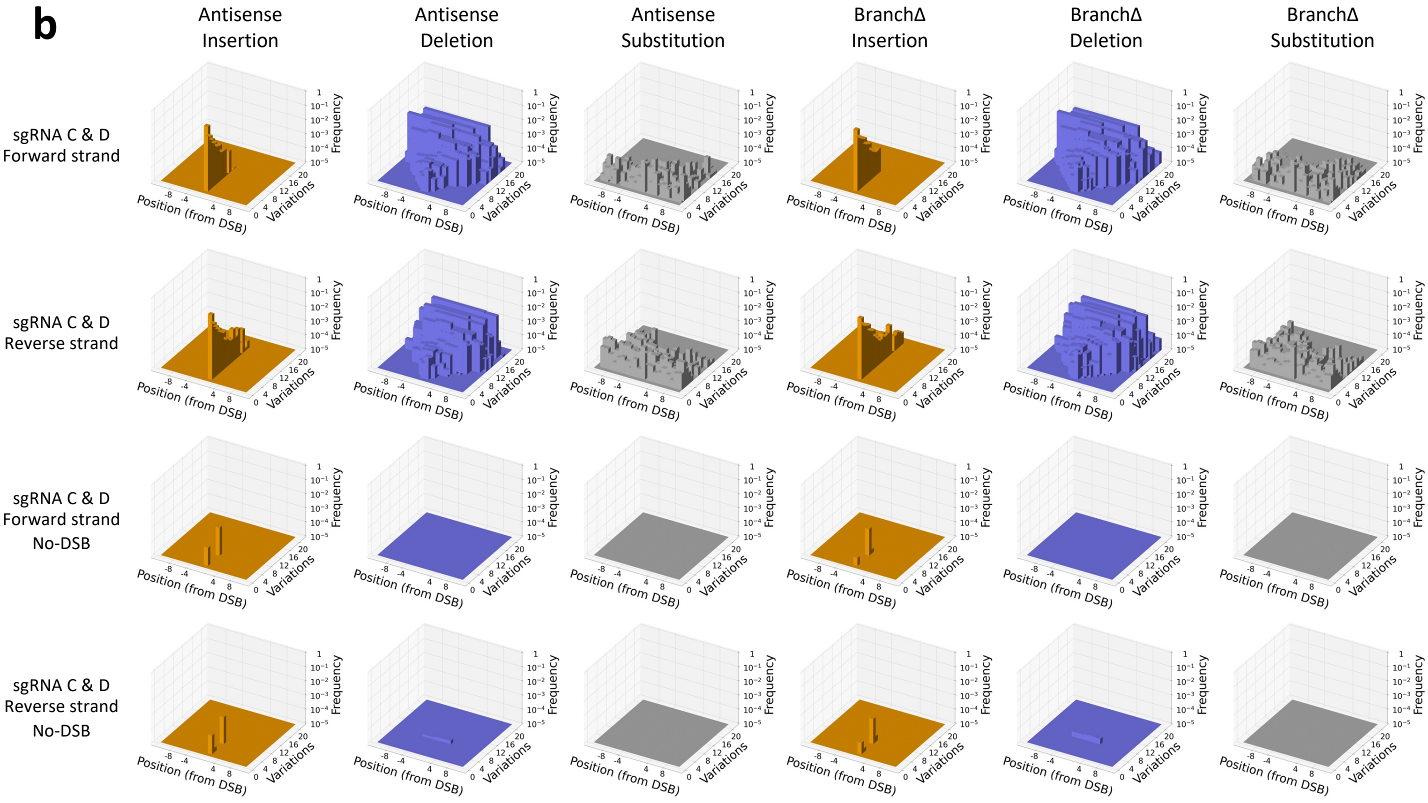

rnh1 rnh201 (*spt3*)

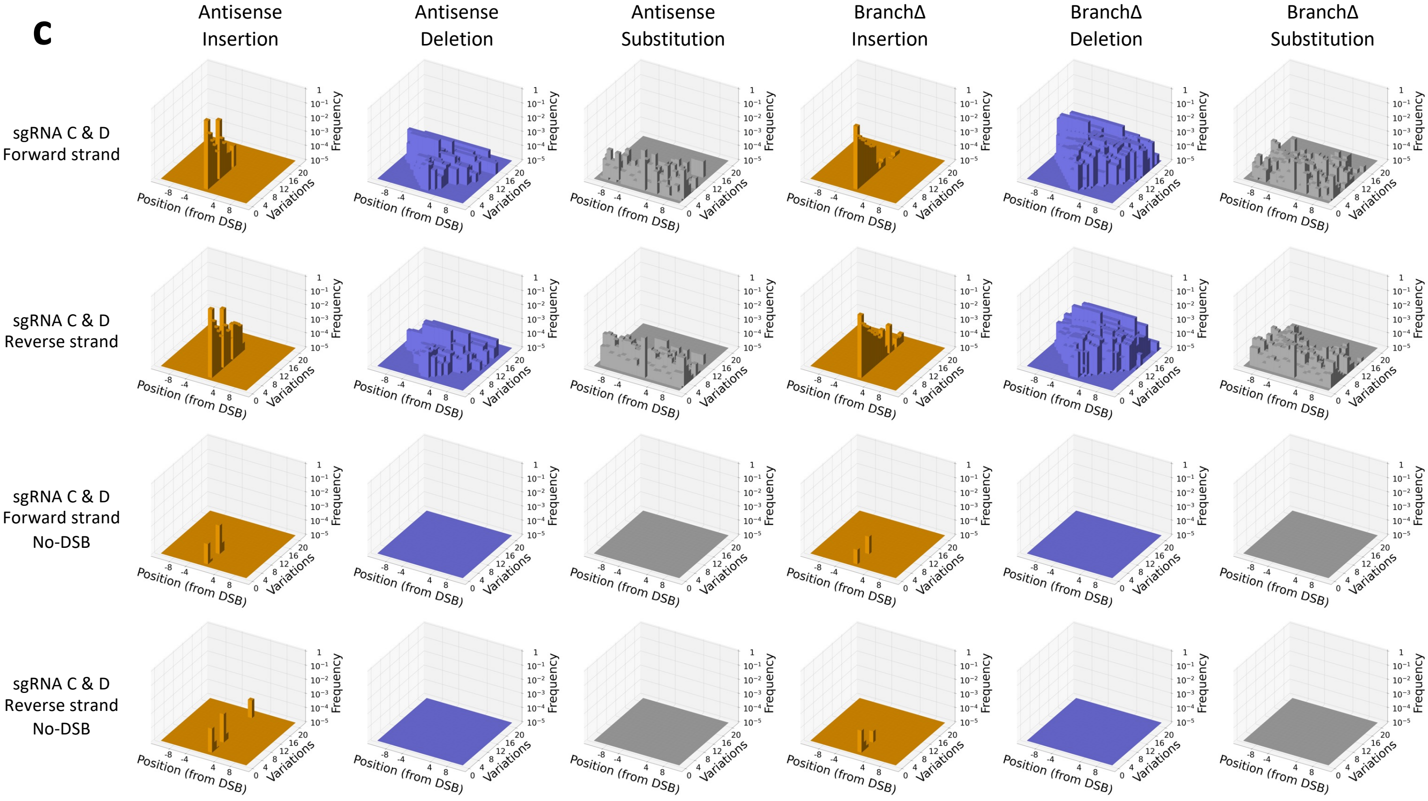

Supplementary Figure 17 | In/dels are sequence variations specific to DSB repair by NHEJ detected in the yeast constructs.

Variation-position histograms showing the distribution of sequence variations in the 20-bp DSB-sequence windows categorized by the position of the variations and the number of variations. The histograms are arranged in a grid: rows specify the sgRNA(s) used to induce the DSB(s), including the No-DSB controls; columns specify the construct and the type of variation (insertion in orange, deletion in blue, or substitution in gray). The x-axis indicates the position of the variations relative to the DSB site on the reference sequence. Figures with reverse-strand data have their x-axis coordinates reversed so that they correspond to forward-strand coordinates. The y-axis indicates the total number of variations in the DSB-sequence windows. The z-axis indicates the total sum of frequencies (log-scale) of DSB-sequence windows with y variations including position x. If a DSB-sequence window has more than one variation (say k) at the same position (which can only happen if the DSB-sequence window contains insertions), this window contributes k-fold to the corresponding z-value. The x, y, z axes have been limited to the ranges $[-10, 10]$, $[0, 20]$, and $[10^{-5}, 1]$, respectively, and values outside these ranges have been cropped. Like in the 2-DSB experiments with sgRNAs C & D, in the No-DSB experiments with sgRNAs C & D the reads were aligned to the 2-DSB reference sequence, which is the sequence of the construct between the two sequencing primers with the nucleotides between the two DSB sites deleted. Since the No-DSB reads had small frequency of gap deletion (i.e. intron pop-out, 0.02% to 1.0%), the reads mostly fail to align with the reference sequence, which caused variations (especially substitutions) to be nearly undetectable in many cases. The variations that are detected in the No-DSB controls are likely in reads with gap removal due to leakiness of the pGAL promoter causing Cas9 to be expressed in the cells. **a,** Variation-position histograms for experiments conducted in the wild-type yeast cells, **b,** in the s*pt3* KO cells (wild-type RNase H cells), and **c**, in the *rnh1 rnh201 spt3* KO cells (*rnh1 rnh201*).

Supplementary Figure 18

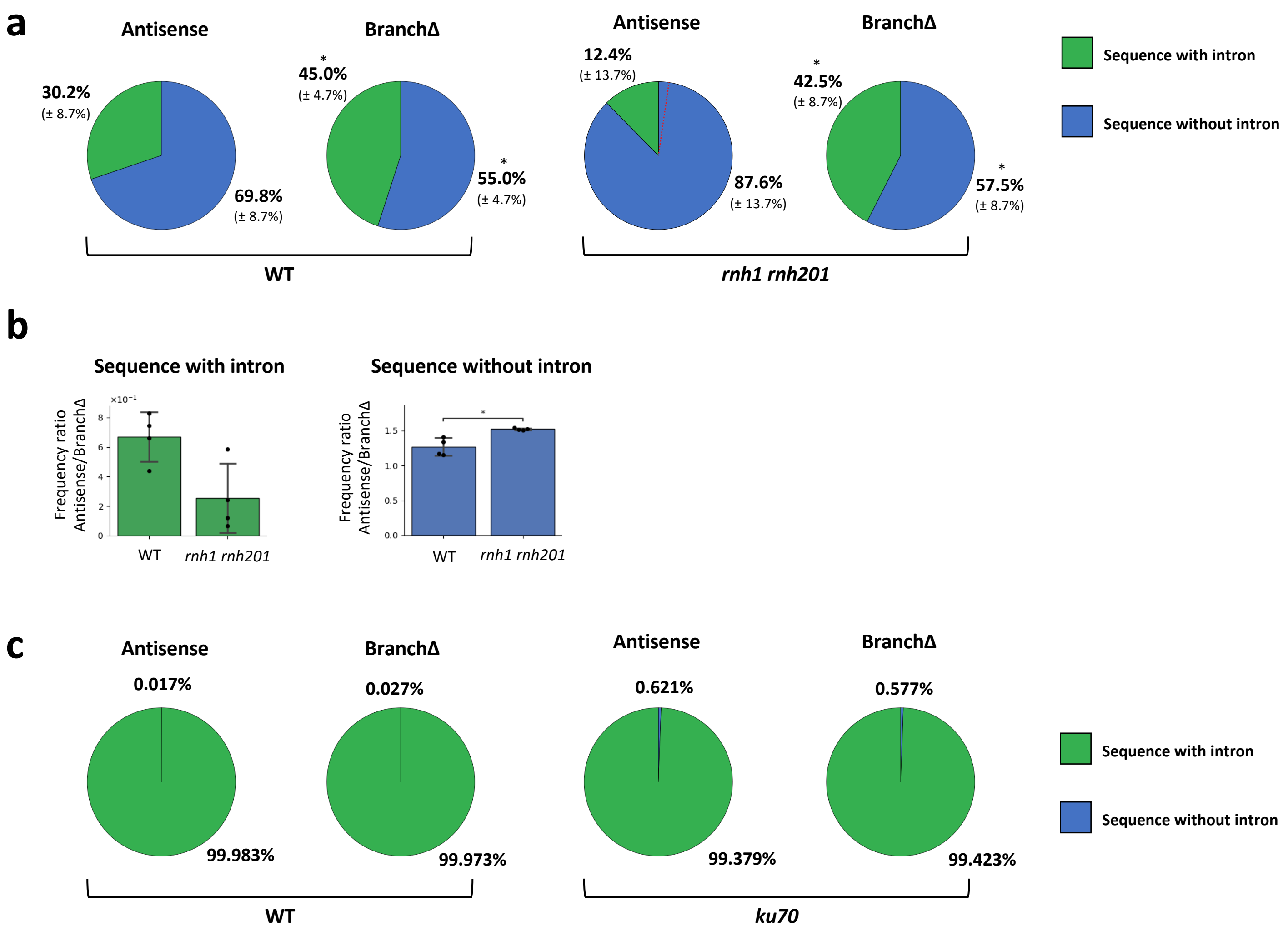

Supplementary Figure 18 | RNA-mediated DSB repair following a DNA double-strand gap is promoted in the absence of RNase H function in yeast cells.

a, Pie charts showing frequencies of sequencing reads displaying intron retention or pop-out following a double-strand gap by the sgRNAs C and D in the Antisense and BranchΔ constructs of yeast *spt3* knock-out (left, WT) and *rnh1 rnh201 spt3* triple knock-out (right, *rnh1 rnh201*) cells. The fraction of sequences containing the intron is shown in green, and the fraction without the intron is shown in blue. Within the blue fraction of sequences without the intron, the red dotted line marks a small fraction of repaired sequences having an identical sequence with the spliced RNA, which could be the result of either NHEJ or RNA-templated DSB repair (R-TDR). The frequencies of the repair events having the same sequence as the spliced RNA are 0.03% for WT, Antisense (± 0.01%), 0.03% for WT, BranchΔ (± 0.04%), 2.29% for *rnh1 rnh201*, Antisense (± 1.15%), and 0.02% for *rnh1 rnh201*, BranchΔ (± 0.02%). Percentages represent an average of 4 repeats with standard deviation in parenthesis; N=4. The percentages of sequences with and without intron are bolded. *, $P = 0.029$ comparing frequencies of the BranchΔ with those of the Antisense construct via the two-tailed Mann-Whitney *U* test. **b,** Antisense/BranchΔ frequency ratios for two types of repaired sequences, with intron (green) and without intron (blue), detected in the sequencing libraries following a double-strand gap by the sgRNAs C and D in the *spt3* knock-out (WT) and *rnh1 rnh201 spt3* triple knock-out (*rnh1 rnh201*) cell types. Plotted data are the mean ± s.d. of the ratios of the 4 biological replicates with the individual values shown as dots; N=4. *, $P = 0.029$ (two-tailed Mann-Whitney *U* test). **c,** Pie charts showing frequencies of sequencing reads displaying intron retention or pop-out for no DSB controls without Cas9 expression (see **Method**) in the Antisense and BranchΔ constructs of yeast WT (left) and *ku70* knock-out (right), N=1. Source data are provided as a Source Data file.
