# Supplementary material for: RNA-mediated double-strand break repair by end-joining mechanisms
Source: Nat Commun. 2024 Sep 11;15:7935. doi: 10.1038/s41467-024-51457-9 (PMC11390984; doi:10.1038/s41467-024-51457-9)
Supplement: Supplementary file 3 — Description of Additional Supplementary Files [file 41467_2024_51457_MOESM3_ESM.pdf]

Description of Additional Supplementary Files

File Name: **Supplementary Data 1**

Description: List of oligonucleotides used in this study.
Names of oligonucleotides used in this study are shown with their sequence in the 5′ to 3′ direction, and corresponding description. *, Phosphorothioate bond; /Cy5/, Cy5 dye; r, ribonucleotide.

File Name: **Supplementary Data 2**

Description: List of synthesized sgRNAs used in this study.
The sgRNAs used in this study are shown with their names, sequences, and description. The m is a chemical modification in a nucleotide provided by Integrated DNA Technologies for the Alt-R CRISPR-Cas9 sgRNA products. The 'r' means a ribonucleotide. *, Phosphorothioate bond.

File Name: **Supplementary Data 3**

Description: *P*-values comparing Sense/BranchΔ frequency ratios of RNA-mediated DNA repair between the HEK-293T RNase H2A wild-type and KO cells by Mann-Whitney *U* test.
The 2 DSBs are generated by sgRNA A and sgRNA B. Two-tailed Mann-Whitney *U* tests were performed (see Methods) to test whether the frequency ratio of Sense/BranchΔ was greater in wild-type (WT) or RNase H2A knock-out (KO) samples. In the Conclusion column: 'NS' means 'not significant'; '* WT' means Sense/BranchΔ is greater in wild-type at $P < 0.05$; '* KO' means Sense/BranchΔ is greater in RNase H2A knockout at $P < 0.05$.

File Name: **Supplementary Data 4**

Description: *P*-values comparing Sense/BranchΔ frequency ratios of RNA-mediated DNA repair between the RNase H1 normal and overexpression by Mann-Whitney *U* test.
The 2 DSBs are generated by sgRNA A and sgRNA B. Two-tailed Mann-Whitney *U* tests were performed (see Methods) to test whether the frequency ratio of Sense/BranchΔ was greater in control (CT) or RNase H1 overexpression (OX) samples. In the Conclusion column: 'NS' means 'not significant'; '* CT' means Sense/BranchΔ is greater in the control at $P < 0.05$; '* OX' means Sense/BranchΔ is greater in RNase H1 overexpression at $P < 0.05$.

File Name: **Supplementary Data 5**

Description: Yeast strains used in this study.

File Name: **Supplementary Data 6**

Description: Categorization of the reads not classified as error-free NHEJ/uncut, NHEJ with in/dels, or MMEJ in the 1-DSB experiments (sgRNA A and sg RNA B) and their corresponding No-DSB controls in wild-type and RNase H2A knock-out (KO) cells.
The Category column shows the five categories: MMEJ-like deletion (exon-exon), MMEJ-like deletion (exon-intron), MMEJ-like deletion (exon-branch) (only for Sense and pCMVΔ experiments, which have the branch site), In/dels shifted ≤ 3 nt from the DSB site, and Unclassified in No-DSB control (reads that appear in high frequency in the No-DSB control also, including in/dels 4 nt or farther away from the DSB site). The Mean and SD show the mean and standard deviation of the repeat experiments (N = 1 for No-DSB controls and N = 4 for all others). The *P*-value shows the two-tailed Mann-Whitney U test comparing the Sense or pCMVΔ construct frequencies to the BranchΔ frequencies (only in the rows for Sense and

pCMVΔ experiments that are not No-DSB control; category MMEJ-like deletion (exon-branch) is omitted since it is not present in BranchΔ; N = 4). Conclusion shows 'NS' when $P \geq 0.05$, otherwise if $P < 0.05$, it shows '* S' if the Sense experiments had higher frequencies, '* B' if the BranchΔ experiments had higher frequencies, or '* C' if the pCMVΔ experiments had higher frequencies. Observe that in all cases the category 'MMEJ-like deletion (exon-exon)' had higher frequencies in Sense and pCMVΔ experiments or were not significantly different, while the categories 'MMEJ-like deletion (exon-intron)' and 'In/dels shifted ≤ 3 nt from the DSB site' had higher frequencies in BranchΔ or were not significantly different. This parallels the pattern observed in the categories MMEJ exon-exon, MMEJ exon-intron, and NHEJ with in/dels, respectively, in the main results.
